# Chimeric iPSC-Derived Lung Organoids Reveal the Minimum Wild-Type Cell Fraction Detectable by the Forskolin-Induced Swelling Assay in Cystic Fibrosis

**DOI:** 10.3390/ijms27219765

**Published:** 2026-10-30

**Authors:** Anna Demchenko, Margarita Lopatina, Irina Panchuk, Aleksandra Nazarova, Anastasiia Ivleva, Elena Amelina, Ekaterina Voronina, Victoria Pozhitnova, Konstantin Kochergin-Nikitsky, Maxim Balyasin, Valeria Kovalskaia, Oksana Ryzhkova, Alexander Lavrov, Svetlana Smirnikhina

**Affiliations:** 1 Research Centre for Medical Genetics, Moskvorechye, 1, 115522 Moscow, Russia; l.pearl.v@gmail.com (M.L.); panchuk_io@mail.ru (I.P.); ag.nazarova@mail.ru (A.N.); nastyaivleva0809@gmail.com (A.I.); vekats@inbox.ru (E.V.); victoriapozhitnova@gmail.com (V.P.); kochnik.ks@gmail.com (K.K.-N.); kovalskaya@med-gen.ru (V.K.); o.ryzhkova@med-gen.ru (O.R.); alexandervlavrov@gmail.com (A.L.); smirnikhinas@gmail.com (S.S.); 2 Research Institute of Pulmonology, 11th Parkovaya Str., 32/4, 105077 Moscow, Russia; eamelina@mail.ru; 3 Scientific and Educational Resource Center, Peoples’ Friendship University of Russia, 117198 Moscow, Russia; max160203@gmail.com

**Keywords:** cystic fibrosis, induced pluripotent stem cells, chimeric organoids

## Abstract

Cystic fibrosis is a life-threatening inherited disease caused by pathogenic variants in the CFTR gene that impair chloride and bicarbonate transport across airway epithelia. Gene editing and cell replacement strategies hold promise for restoring CFTR function, yet they inevitably generate heterogeneous cell populations in which only a fraction of cells carry a corrected allele, raising the question of how large this fraction must be for functional rescue to be detectable and whether the forskolin-induced swelling (FIS) assay is sensitive enough to capture partial correction. To address this, we generated chimeric iPSC-derived lung organoids by co-culturing GFP-labeled wild-type airway basal cells with unlabeled F508del-homozygous cystic fibrosis basal cells at seven predefined ratios (0–100% wild-type), verifying the actual wild-type fraction by flow cytometry and quantifying 24-h forskolin-induced swelling as the area ratio. Chimeric organoids showed a dose-dependent increase in swelling with rising wild-type fractions, with a statistically significant FIS response first resolved at approximately 6% wild-type cells in the pooled analysis. Separately, ROC analysis of well-level data identified two area-ratio cutoffs for classifying organoid populations—1.16 (80% sensitivity, 80% specificity) and 1.21 (98% specificity, 70% sensitivity)—which the fitted mean response reaches at approximately 14% and 34% wild-type cells, respectively. The minimum statistically detectable fraction and the classification cutoffs are distinct quantities derived from different analyses and are not interchangeable; both describe the performance of the FIS assay in heterogeneous organoids rather than thresholds for therapeutic benefit and provide a practical reference framework for designing and interpreting gene editing and cell therapy studies in cystic fibrosis.

## 1. Introduction

Cystic fibrosis (CF) is an autosomal recessive multisystem disease caused by pathogenic variants in the CFTR gene, which encodes an epithelial anion channel mediating chloride and bicarbonate transport across apical membranes [1]. Defective CFTR function leads to dysregulated ion and water transport, dehydration and hyperviscosity of epithelial secretions, impaired mucociliary clearance, and chronic airway infection and inflammation that ultimately drive progressive structural lung damage and respiratory failure. Despite improvements in symptomatic care and organ-specific management, lung disease remains the main cause of morbidity and mortality in patients with CF.

The emergence of genome editing technologies, including CRISPR-Cas9, base editing, and prime editing, has opened the possibility of directly correcting pathogenic CFTR alleles in patient-derived cells, offering a potentially curative therapeutic approach [2]. However, gene editing protocols do not achieve complete correction in all cells; rather, they generate heterogeneous populations containing a mixture of successfully edited CFTR-wild-type cells and uncorrected CFTR-mutant cells. This cellular mosaicism underscores the importance of determining the threshold proportion of CFTR-functional cells required to restore physiologically meaningful ion transport and achieve clinical benefit.

Multiple lines of evidence from both experimental models and clinical observations suggest that full restoration of CFTR function in every cell may not be required. Studies using co-cultures of normal and CF airway epithelial cells measured by Ussing chamber electrophysiology have demonstrated that mixed populations containing as little as 10–20% wild-type cells can restore CFTR-dependent chloride transport to approximately 40–70% of normal levels [3,4]. Similarly, Zhang et al. demonstrated that viral vector-mediated delivery of functional CFTR to approximately 25% of ciliated surface epithelial cells in a CF human airway epithelium model was sufficient to restore normal rates of mucus transport [5]. In radioactive chloride efflux assays, the sensitivity threshold for detecting CFTR activity in mixed populations was reached at ≥25% normal cells, although modest functional gains were detectable even at 1% wild-type cells in transepithelial measurements [6]. Clinical correlations reinforce these experimental thresholds. Patients carrying splice variants that permit residual production of normal CFTR mRNA at levels as low as 5–8% of wild-type exhibit significantly milder pulmonary phenotypes compared to individuals with F508del homozygotes, suggesting that even modest restoration of CFTR expression can substantially ameliorate disease severity [7]. Furthermore, CFTR modulator therapy shows that partial pharmacological restoration of CFTR function translates into measurable clinical benefit: in people with cystic fibrosis carrying at least one G551D allele, ivacaftor improved percent-predicted forced expiratory volume in 1 s (FEV_1_) by 10.6 percentage points relative to placebo, lowered sweat chloride by 48.1 mmol/L, and reduced the risk of pulmonary exacerbations by 55% over 48 weeks [8]. Collectively, these data converge on a working hypothesis that correction of a relatively small fraction of epithelial cells—in the range of 5–25%—may suffice to confer significant therapeutic benefit [9].

Forskolin-induced swelling (FIS) of patient-derived intestinal organoids has emerged as a gold-standard functional assay for evaluating CFTR activity in three-dimensional epithelial models [10]. More recently, airway organoids have gained increasing attention as a complementary FIS platform, given that the lungs represent the primary organ affected in CF and airway-derived models more closely recapitulate the disease-relevant epithelial environment [11]. In the FIS assay, activation of intracellular cAMP signaling by forskolin stimulates CFTR-mediated chloride and fluid secretion into the organoid lumen, causing rapid luminal expansion that can be quantified by time-lapse imaging. FIS is now widely used for personalized medicine approaches, including ex vivo drug testing and functional validation of rare CFTR variants, and it represents a highly relevant readout for assessing the efficacy of gene editing strategies applied to airway progenitor cells. A key unresolved question is the sensitivity and dynamic range of the FIS assay in heterogeneous cell populations, particularly in the context of gene editing, where only a fraction of cells may carry a corrected CFTR allele. While several studies using epithelial monolayers or animal models have suggested that a relatively small proportion of CFTR-positive cells (on the order of 5–20% of the epithelium) may be sufficient to substantially improve chloride transport, a systematic calibration of FIS against a known fraction of CFTR-competent cells has not been performed. Without such calibration, it is difficult to interpret negative or modest FIS responses after gene editing because they may reflect either low correction efficiency or intrinsic assay limitations in detecting small functional gains.

Induced pluripotent stem cells (iPSCs) provide a renewable and genetically defined source of human cells for disease modeling and therapeutic development in CF. iPSCs can be differentiated into induced airway basal cells (iBCs) and induced lung organoids (iLOs) that recapitulate key structural and functional features of the native airway epithelium, including CFTR-dependent ion transport [12]. By combining iBCs with wild-type CFTR and F508del-homozygous iBCs at controlled ratios and measuring the resulting FIS response in chimeric iLOs, it becomes possible to establish both the smallest wild-type fraction at which the FIS assay resolves a response from CF-only controls and the larger fractions at which the mean response reaches the area-ratio cutoffs used to classify organoids as CFTR-functional.

In this study, we established a chimeric iLOs model composed of iBCs carrying both wild-type CFTR (Mak-f line, WT/WT genotype, labeled with lentiviral GFP) and pathogenic CFTR variants (P1L5 and P7L2 lines, F508del/F508del genotype). Chimeric iLOs were generated from defined mixtures ranging from 0% to 100% Mak-f cells; the actual proportion of GFP-positive cells in each organoid population was verified by flow cytometry, and FIS was quantified after 24 h of forskolin exposure. This approach yielded a dose–response relationship between the fraction of CFTR-wild-type cells and organoid functional activity, providing the first calibration of FIS assay sensitivity in the context of chimeric iLOs.

## 2. Results

### 2.1. Generation and Characterization of GFP-Labeled iPSCs

**Figure 1:**
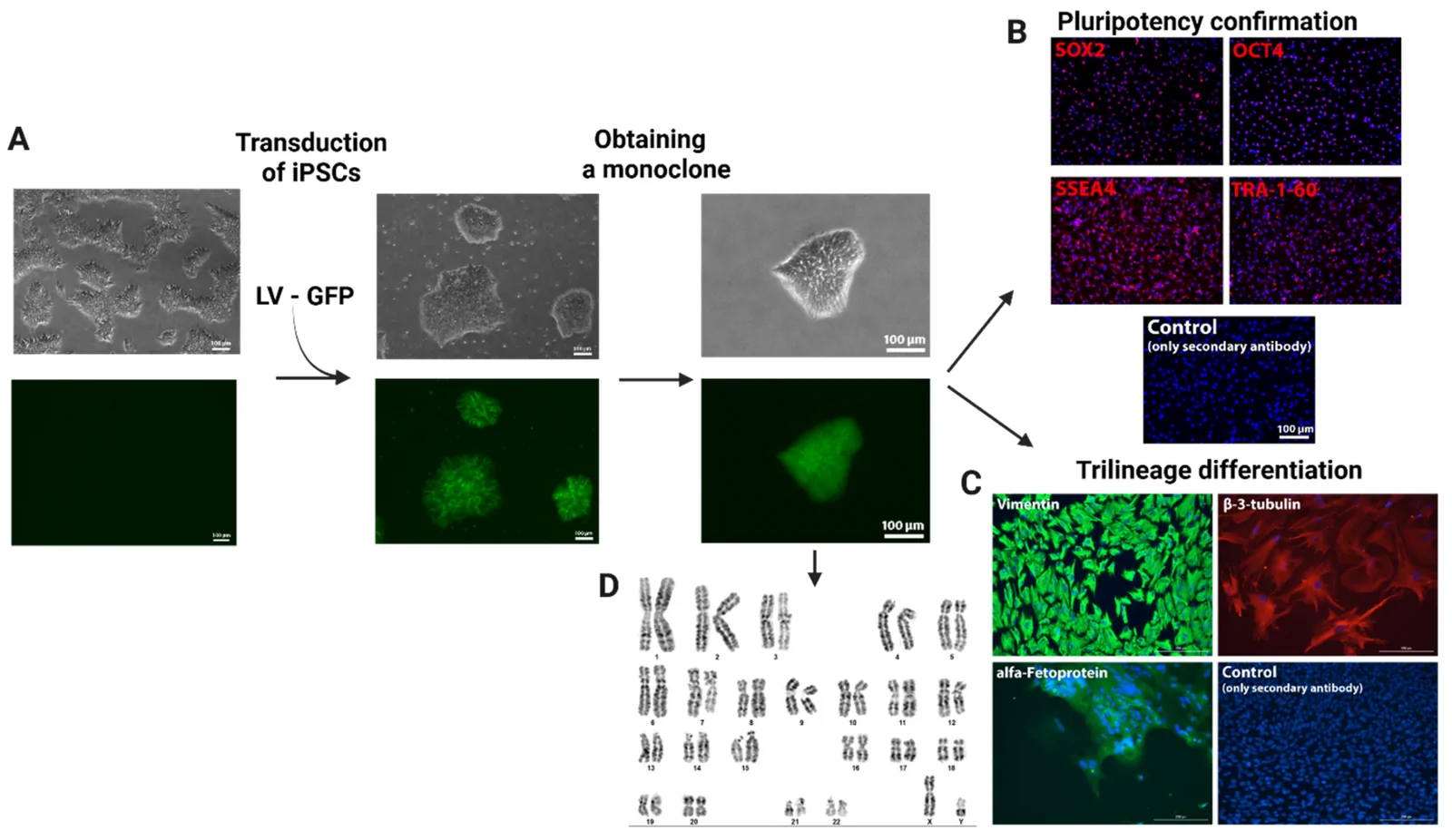
Generation and characterization of the monoclonal GFP-positive iPSC line: (**A**) Morphology and GFP expression in the established monoclonal iPSC line (Mak-f) transduced with the LV-GFP vector. Scale bar, 100 µm. (**B**) Immunofluorescence analysis confirming the maintenance of pluripotency, indicated by expression of core pluripotency markers (TRA-1-60, SOX2, SSEA4, and OCT4). Scale bar, 100 µm. (**C**) Spontaneous in vitro trilineage differentiation of the GFP-positive iPSC clone via embryoid body formation. Representative immunostaining images confirm successful differentiation into the three embryonic germ layers, showing positive expression of β-III tubulin (ectoderm), α-fetoprotein (endoderm), and vimentin (mesoderm). Nuclei were counterstained with DAPI. Scale bar, 200 µm. (**D**) Karyotype of the GFP-positive iPSC clone.

A GFP-positive monoclonal iPSC line was established from the parental Mak-f line (WT/WT in CFTR) by lentiviral transduction with a vector encoding GFP, achieving a transduction efficiency of 97%. A GFP-positive monoclonal line was subsequently isolated and subjected to further characterization (Figure 1A). The obtained monoclonal cell line was maintained in culture for several months, during which the cells continued to exhibit stable GFP fluorescence. Immunofluorescence staining demonstrated that the monoclonal iPSC line retained pluripotent properties, as indicated by positive expression of the pluripotency markers TRA-1-60, SOX2, SSEA4, and OCT4 (Figure 1B). To further characterize the monoclonal line, trilineage differentiation was induced via embryoid body formation. Immunostaining for germ layer-specific markers—vimentin (mesoderm), β-III-tubulin (ectoderm), and alpha-fetoprotein (endoderm)—confirmed successful spontaneous differentiation following LV-GFP modification (Figure 1C). At the 5th passage, the monoclonal iPSC line showed a normal 46,XY karyotype (Figure 1D). The obtained GFP^+^ iPSC monoclonal line was subsequently used for the generation of iBCs and chimeric iLOs.

Analysis of the GFP-positive iPSC Mak-f line revealed several lentiviral integration sites. Integration events included intergenic regions on chromosomes 4 (chr4:179,346,287) and 3 (chr3:68,567,933) and intronic integration sites of the following genes: *AP2A1* (chr19:49,787,951), *LINC01118* (chr2:46,760,173), and *SCAI* (chr9:124,972,549). None of the targeted loci are established proto-oncogenes commonly implicated in insertional mutagenesis, and none has a documented role in CFTR biogenesis, trafficking, or gating.

To test directly whether proviral insertion had perturbed transcription at these loci, steady-state transcript levels were quantified by RT-qPCR in the GFP^+^ clone and in the untransduced parental line (primer sequences, Table S1; quantification, Table S2). *SCAI* showed no reduction in the clone and was more abundant there (ΔΔCt −2.95 ± 1.02, 7.7-fold). *AP2A1* amplified at the limit of detection in both lines, and the two were not informative (ΔΔCt −0.29 ± 1.16, 1.2-fold). *LINC01118* was less abundant in the clone (ΔΔCt +2.14 ± 0.40, 4.4-fold); as the provirus occupies a single allele, complete silencing of that allele could reduce a transcript by at most one cycle, and a difference of this size argues against a cis-effect without excluding one. This ceiling applies only to reduced expression and does not account for the 7.7-fold increase in *SCAI*, which a cis-effect of the insertion cannot produce and which clonal or trans effects could; the measurements are therefore not definitive either for or against an integration-related effect at these loci.

Organoids composed entirely of GFP^+^ Mak-f cells swelled to the extent expected for unmodified wild-type iLOs [13], arguing against a functionally relevant perturbation in the labeled line.

### 2.2. Generation of GFP-Positive iBCs and Chimeric iLOs

**Figure 2:**
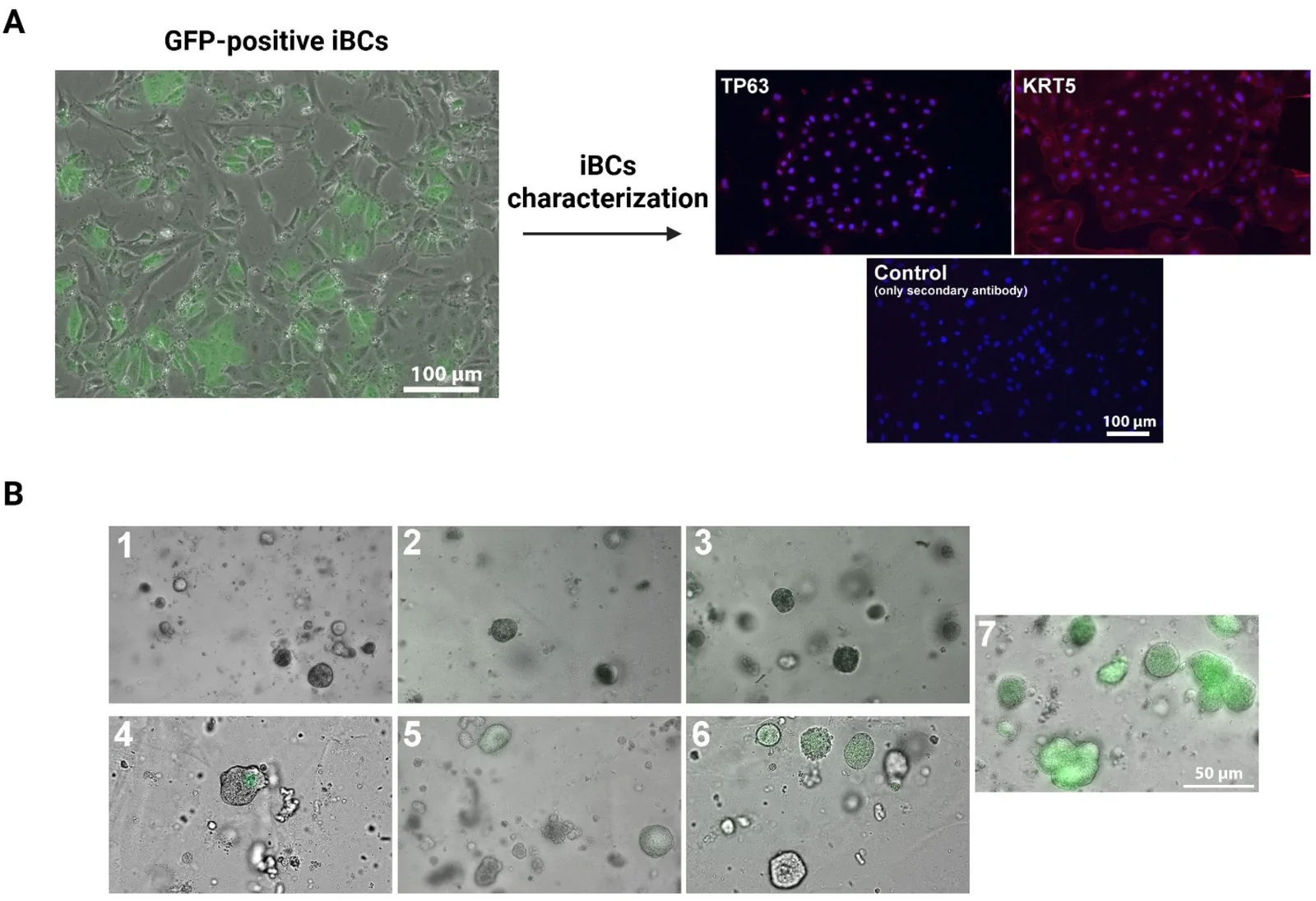
Generation of GFP-labeled iBCs and chimeric iLOs: (**A**) Phase-contrast and fluorescence images of GFP-positive iBCs (Mak-f line) stained for basal cell markers. Nuclei were counterstained with DAPI (blue). Scale bar, 100 µm. (**B**) Representative images of chimeric iLOs (P1L5 and Mak-f lines) on day 7 after formation. Seven experimental groups of iLOs were assembled with the planned ratios of cells as follows: (1) P1L5—100%, Mak-f—0%; (2) P1L5—99%, Mak-f—1%; (3) P1L5—95%, Mak-f—5%; (4) P1L5—90%, Mak-f—10%; (5) P1L5—85%, Mak-f—15%; (6) P1L5—80%, Mak-f—20%; (7) P1L5—0%, Mak-f—100%. Scale bar, 50 µm.

Following the generation of the GFP-positive iPSC monoclonal line, we performed differentiation into iBCs. Upon completion of the differentiation process, the cells maintained GFP expression, and their basal cell phenotype was confirmed by the presence of TP63 and KRT5 markers (Figure 2A). To exclude the possibility of aberrant differentiation towards alternative epithelial lineages, iBCs were additionally stained for markers of intestinal progenitor cells (MUC2, LGR5) and hepatic progenitor cells (AFP) (list of primary antibodies provided in Table S3). No immunoreactivity was detected for any of these markers, confirming that the differentiation protocol produced a lung-specific basal cell phenotype without contamination from non-airway lineages (Figure S1). The proliferative behavior of the GFP^+^ monoclonal line did not differ from that of the untransduced parental Mak-f population: over 96 h, the two populations completed 3.5 ± 0.3 and 3.4 ± 0.4 population doublings, respectively (*p* = 0.643), corresponding to population doubling times of 27.5 ± 2.1 h and 28.8 ± 3.4 h (*p* = 0.621; Figure S2).

Next, we established a chimeric iLO model by combining GFP-positive Mak-f iBCs with GFP-negative CF iBCs derived from F508del/F508del patient lines (P1L5 or P7L2). Chimeric iLOs were assembled using predefined input ratios of Mak-f cells (0%, 1%, 5%, 10%, 15%, 20%, and 100%). Representative bright-field and fluorescence images demonstrated the successful incorporation of GFP-positive cells into the resulting organoid structures (Figure 2B). The images confirm that GFP-positive Mak-f cells integrated into the chimeric organoids rather than forming distinct, separate organoids, which allows for a reliable correlation of functional readouts with the proportion of CFTR-competent Mak-f cells. To characterize the cellular composition of iLOs, organoid immunofluorescence staining was performed (list of primary antibodies provided in Table S4). Organoids contained AQP1- and PDPN-positive lung epithelial cells, as well as differentiated epithelial cell types, including Club cells (SCGB3A2^+^) and goblet cells (MUC5AC^+^), suggesting that the iBC-derived chimeric iLOs display features of a multicomponent epithelial architecture partially resembling airway and distal lung epithelium (Figure S3). GFP immunostaining confirmed the presence of Mak-f WT/WT-GFP-derived cells within chimeric iLOs, whereas control organoids composed exclusively of Mak-f WT/WT-GFP cells displayed uniform GFP signal throughout the epithelium, further validating the successful incorporation of the wild-type cell fraction into the chimeric organoid structures (Figure S4).

### 2.3. Dose-Dependent Restoration of CFTR Function in Chimeric iLOs

To determine whether the introduction of CFTR WT/WT Mak-f iBCs into CF organoid cultures restores CFTR-dependent function in a dose-dependent manner, we measured FIS in chimeric iLOs across seven Mak-f cell fractions (0–100%) in combination with either P1L5 or P7L2 CF iBCs. The organoid area ratio after 24-h forskolin exposure served as the functional readout. In all experimental series, the actual proportion of GFP-positive Mak-f cells in each chimeric iLO population—verified by flow cytometry after organoid dissociation—diverged substantially from the planned seeding ratios, confirming that empirical quantification of cell composition is essential for the accurate interpretation of functional outcomes in chimeric organoid models.

**Figure 3:**
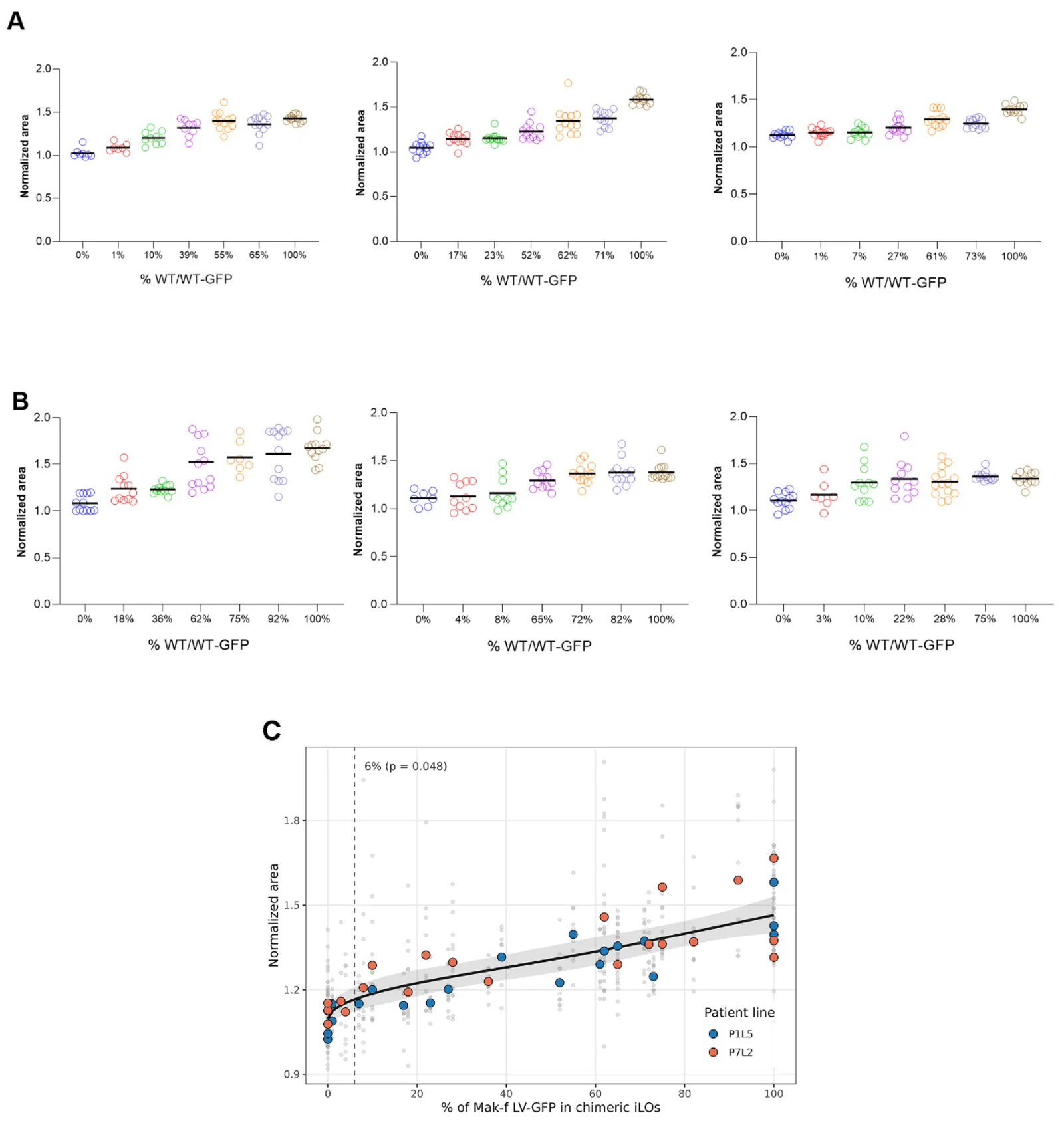
Dose-dependent restoration of CFTR-mediated swelling in chimeric iLOs. Chimeric iLOs were generated by combining CFTR-competent Mak-f cells (WT/WT-GFP) with CF patient-derived P1L5 or P7L2 cells (F508del/F508del), and FIS was measured as the organoid area ratio after 24 h of exposure to 10 µM forskolin. Each symbol is one well, summarized by the geometric mean of the per-organoid area ratios of the organoids it contained; approximately 20–150 organoids were analyzed per condition and experiment: (**A**) FIS response in chimeric iLOs composed of Mak-f and P1L5 cells. (**B**) FIS response in chimeric iLOs composed of Mak-f and P7L2 cells. The x-axis indicates the actual percentage of GFP-positive Mak-f cells integrated into the organoids, as quantified by flow cytometry; data are presented as mean ± SD. (**C**) The same experiments analyzed together on the measured area-ratio scale, without normalization. Grey symbols, individual wells (n = 479); colored symbols, experimental conditions (n = 42), colored by CF patient line, plotted at the actual percentage of GFP-positive Mak-f cells determined by flow cytometry of dissociated organoids immediately after the FIS assay; line and shaded band, estimated marginal mean and its 95% CI from the linear mixed-effects model (fixed effects, CF line and a natural cubic spline in the square root of the GFP-positive percentage; random intercepts, experiment and condition within experiment), averaged over the two CF lines and back-transformed from the log scale; dashed vertical line, the smallest fraction of GFP-positive Mak-f cells at which the modeled mean differs from the CF-only controls (6%, *p* = 0.048).

Across three independent experiments, chimeric iLOs composed of GFP-positive Mak-f (CFTR WT/WT) and P1L5 (CFTR F508del/F508del) cells showed a dose-dependent increase in FIS response with rising actual fractions of Mak-f cells (Figure 3A). Representative bright-field images of chimeric iLOs (Mak-f WT/WT-GFP + P1L5 F508del/F508del) at t = 0 h and t = 24 h after forskolin addition for all seven experimental groups are provided in Figure S5. In the 0% Mak-f control conditions, baseline area ratios were uniformly low (1.03–1.13), reflecting the absence of functional CFTR activity (Table S5). In the per-experiment comparisons shown in Figure 3A,B, an increase above the CF-only controls was first apparent when the actual GFP-positive fraction reached approximately 10–27% (producing area ratios of 1.15–1.20). As the actual proportion of Mak-f cells increased further to 52–73%, the chimeric organoids exhibited robust swelling responses (area ratios 1.23–1.40) that closely approached the activity levels observed in the 100% wild-type Mak-f controls (area ratio ~1.43). A similar dose-dependent restoration of CFTR function was observed across three independent experiments using P7L2 (CFTR F508del/F508del) (Figure 3B). Some inter-experimental variability in the absolute swelling magnitude of the 100% Mak-f control was observed, which is typical for iPSC-derived organoid systems. Importantly, the dose-dependent relationship between the fraction of CFTR-competent cells and the FIS response was consistently reproduced across all independent experiments and both patient-derived lines. Swelling responses in this background were first detected at actual Mak-f fractions of approximately 10–18% (area ratios 1.24–1.30). Higher actual fractions of CFTR-competent cells drove progressive enhancement of the FIS response, culminating in area ratios of up to 1.67 in the 100% Mak-f reference organoids. In the per-experiment comparisons, a rise above the CF-only controls was thus first apparent at actual fractions of 10–27% CFTR-competent cells across both patient-derived lines. This is a descriptive observation within individual experiments rather than a statistical threshold; the pooled analysis below estimates the smallest fraction at which a response is resolved.

Analyzing all six experiments together in a linear mixed-effects model, chimeric iLOs showed a graded, dose-dependent increase in FIS with rising actual fractions of CFTR-competent Mak-f cells (F(3, 33.5) = 42.06, *p* < 0.001), the fitted response rising monotonically over the observed range, and no difference was detected between the P1L5 and P7L2 backgrounds (F(1, 4.0) = 2.78, *p* = 0.17). Of the variance not explained by the fixed effects, 20% was between conditions, 2% between experiments, and the remaining 78% between wells within a condition. The smallest fraction at which the modeled mean differed from the CF-only controls was 6% GFP-positive Mak-f cells (fold change 1.063, 95% CI 1.0004–1.1303, *p* = 0.048); below 6%, no difference was detected (all *p* >= 0.054) (Figure 3C).

**Figure 4:**
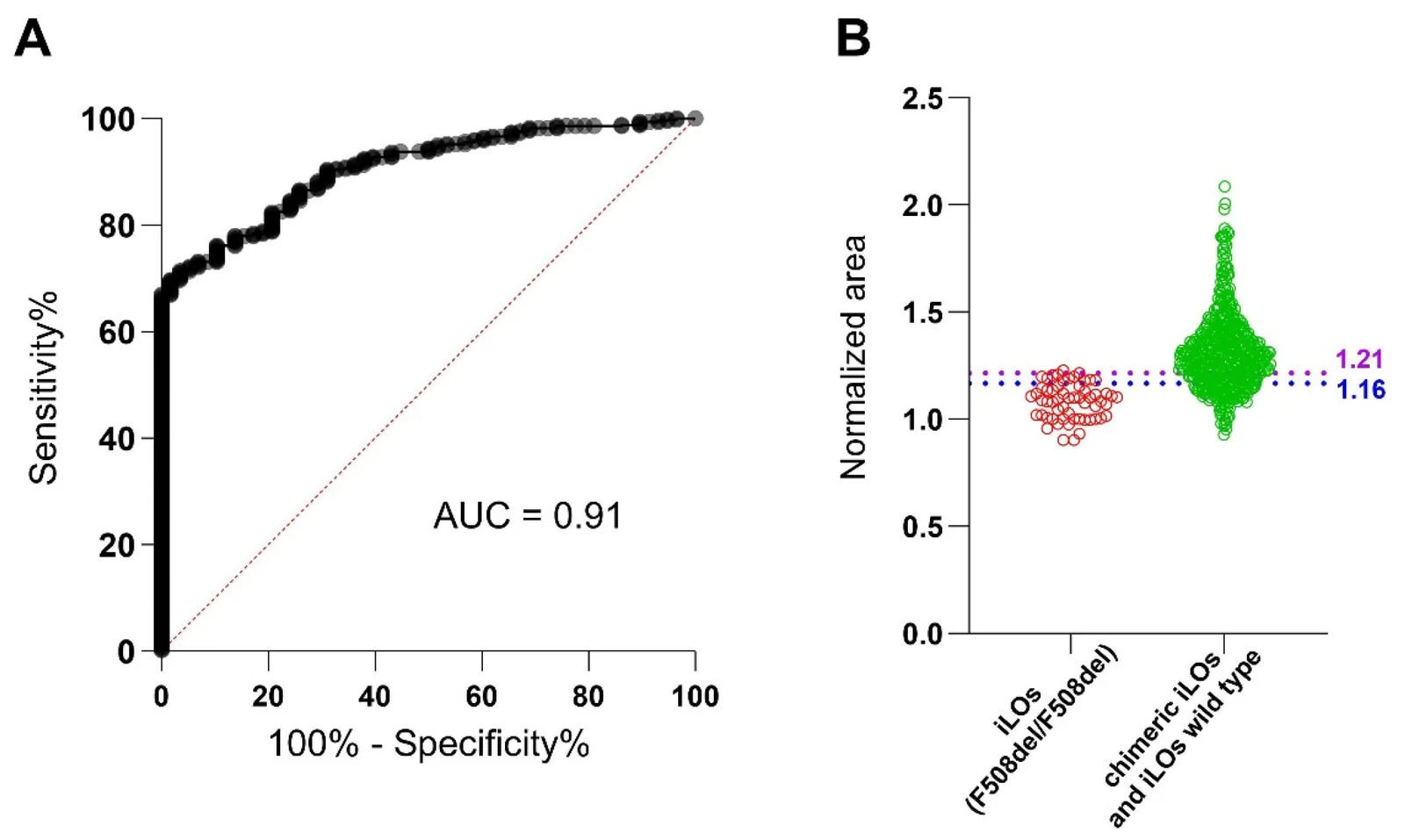
ROC analysis defining experimental FIS cutoffs for detection of CFTR-dependent functional rescue in chimeric iLOs: (**A**) ROC curve generated from pooled FIS data across all chimeric iLO experiments. (**B**) The two experimental FIS cutoffs identified by ROC analysis: a sensitive cutoff of 1.16 (80% sensitivity, 80% specificity) and a stringent cutoff of 1.21 (98% specificity, 70% sensitivity). The fitted mean area ratio reaches these cutoffs at approximately 14% and 34% CFTR-competent cells, respectively. ROC analysis was performed on well-level geometric means of organoid area ratios, with wells of the 0% Mak-f conditions serving as negative cases and all wells of the chimeric and 100% Mak-f conditions as positive cases.

To define quantitative thresholds for FIS-based detection of CFTR functional rescue, a ROC analysis was performed across all pooled chimeric iLO experiments (Figure 4A). Two empirically derived FIS cutoffs were identified (Figure 4B). The first threshold, an area ratio of 1.16 (corresponding to 16% organoid swelling), yielded 80% sensitivity and 80% specificity for discriminating wells containing a WT Mak-f component from CF-only wells; the fitted mean response reached 1.16 at ≈14% actual CFTR-competent Mak-f cells in the chimeric organoid population. The 1.03–1.13 range quoted above refers to condition means, whereas the ROC analysis operates on individual wells, which scatter around those means (Table S5); approximately one well in five from the 0% conditions therefore exceeded 1.16, which is what the 80% specificity of this cutoff records. The second, more stringent threshold—an area ratio of 1.21—achieved 98% specificity at 70% sensitivity, essentially eliminating false-positive responses while still detecting approximately 70% of truly rescued organoids. The fitted mean response reached 1.21 at ≈34% actual CFTR-competent cells.

Collectively, these findings demonstrate that the FIS assay in chimeric iLOs reliably detects a dose-dependent and statistically robust signal consistent with CFTR-dependent functional rescue. The mean response reaches the empirically derived area-ratio cutoffs of 1.16 and 1.21 at approximately 14% and 34%, respectively.

The 24 h endpoint used throughout was examined empirically in a separate, single-plate time-course experiment in which chimeric iLOs at 0% Mak-f, a planned 15% Mak-f fraction, and 100% Mak-f were imaged every 2 h from 0 to 24 h after forskolin addition, the area ratio of each tracked organoid being referred to its own area at t = 0 h (170 organoids from 12 wells per condition, tracked at all thirteen timepoints; Figure S6). Swelling increased monotonically in all three conditions and was still increasing at the end of the window, so every earlier timepoint recorded a smaller response than 24 h. The absolute separation between the 100% and the 0% condition widened from 0.156 area-ratio units at 8 h to 0.268 at 24 h, while the spread between organoids within a condition widened over the same interval. The endpoint was adopted as the longest interval examined, at which swelling was still rising in every condition. The absolute values of this experiment are not comparable with those of the endpoint experiments: the 0% condition reached 1.195 ± 0.239 at 24 h, above the 1.03–1.13 range of the CF-only condition means reported above, and the values here are geometric means taken directly across tracked organoids on a single unreplicated plate imaged thirteen times rather than condition means of well-level geometric means. The area-ratio cutoffs of 1.16 and 1.21 were derived from well-level data of the endpoint experiments and do not apply to them. The experiment was analyzed descriptively, contributed neither to the linear mixed-effects model nor to the ROC analysis, and supports the choice of endpoint only for the conditions and the interval tested.

## 3. Discussion

This study calibrates the FIS assay in chimeric iLOs composed of defined proportions of CFTR-competent and CF cells. By combining GFP-labeled wild-type (Mak-f, WT/WT) with patient-derived CF (P1L5 and P7L2, F508del/F508del) at controlled ratios and verifying the actual cell composition by flow cytometry, a dose–response relationship between the fraction of CFTR-functional cells and organoid swelling was established. Although the FIS assay has become a gold-standard readout for CFTR activity in intestinal and airway organoids, its sensitivity and dynamic range in heterogeneous cell populations—the context created by gene editing or cell replacement—had not been calibrated against known proportions of CFTR-competent cells in a three-dimensional airway model.

The dose–response relationship observed in chimeric iLOs is broadly consistent with, yet extends, previous findings obtained in two-dimensional co-culture systems. Dannhoffer et al. used primary human bronchial epithelial co-cultures and Ussing chamber electrophysiology to demonstrate that as few as 10% of non-CF cells were sufficient to functionally correct Cl^−^ and Na^+^ transport properties and to reduce IL-8 secretion in CF monolayers [3]. In a complementary approach, Illek et al. compared the sensitivity of radioactive chloride (^36^Cl) efflux with Ussing chamber analysis in mixed populations of normal (16HBE14o^−^) and CF (CFBE41o^−^) airway epithelial cell lines [6]. They showed that the Ussing chamber detected cAMP-dependent Cl^−^ currents in mixtures containing as few as 1% wild-type cells, whereas the ^36^Cl efflux assay was reliable only when ≥25% of the cells were normal; at 10% wild-type cells, Ussing analysis revealed 40–50% of normal cAMP-dependent Cl^−^ transport. In the chimeric iLO system described here, the mixed-effects analysis of all six experiments resolved a response from approximately 6% wild-type cells, whereas in the per-experiment comparisons a response was first apparent at 10–27%. The ROC analysis operates on the area ratio rather than on the wild-type fraction: it yielded a cutoff of 1.16 (80% sensitivity, 80% specificity), and the fitted mean response reaches this cutoff at ≈14% CFTR-competent cells. These values span the 10–25% range reported in earlier monolayer studies but extend the finding to a three-dimensional, iPSC-derived airway model that more closely recapitulates the architecture and cellular composition of the native airway epithelium. The stringent cutoff of 1.21 used here coincides with the value previously applied in non-chimeric iLOs to discriminate CFTR-functional from CFTR-deficient organoids during CFTR modulator testing [13]. This coincidence should not be read as independent corroboration because the ROC analysis evaluated custom thresholds alongside the value maximizing Youden’s J statistic, so the agreement is in part a consequence of how the candidate cutoffs were specified. What the chimeric model adds to the earlier work is an estimate of the fraction of CFTR-competent cells at which the mean response reaches this cutoff.

A consistent feature of the chimeric model was the systematic divergence between the nominal seeding ratios and the cell composition actually measured in mature organoids. GFP-positive wild-type cells were over-represented relative to the input ratio across all six independent experiments and both CF donor lines. Several non-exclusive mechanisms could produce it. Organoids arise clonally from a minority of seeded basal cells, so even a modest difference in colony-forming efficiency at the moment of embedding is translated directly into a difference in the number of founder clones and then amplified by clonal expansion. F508del-CFTR is retained in the endoplasmic reticulum and imposes chronic ER stress, and cells carrying it show altered proliferation and impaired wound closure, while loss of apical CFTR alters intracellular and luminal pH and bicarbonate handling—processes directly relevant to lumen formation in a three-dimensional matrix [14,15]. These remain hypotheses: the design cannot establish the cause. Because the wild-type component of every chimera is a single lentivirally transduced, FACS-sorted, clonally expanded subline of one donor, whereas the CF components derive from two different donors, genotype-dependent effects on organoid-forming capacity cannot be separated from donor- or clone-dependent ones. That the enrichment occurred with both CF donors argues against a peculiarity of one CF line, but the wild-type side is represented by a single clone, and a clone-specific effect cannot be excluded. The GFP^+^ subline and the untransduced parental population showed indistinguishable population doublings and doubling times over 96 h (Figure S2), which excludes a general growth advantage acquired during cloning but not a difference in colony-forming efficiency at embedding. Separating genotype from donor and clone would require isogenic corrected and uncorrected lines or a reciprocal design in which the reporter is carried by the CF component.

The three values reported here are distinct quantities and are not interchangeable. Approximately 6% is the minimum statistically detectable fraction: the smallest wild-type fraction at which the pooled model resolved a difference from CF-only controls (fold change 1.063, 95% CI 1.0004–1.1303, *p* = 0.048). It is therefore a property of the assay and of the precision of this dataset, and it did not reproduce within single experiments, in which a response first became apparent at 10–27%. The fitted mean area ratio reaches the ROC-derived cutoffs of 1.16 (80% sensitivity, 80% specificity) and 1.21 (98% specificity, 70% sensitivity) at approximately 14% and 34% wild-type cells, respectively.

This distinction matters when these values are set against clinical data. Ramalho et al. reported that ~5–8% of normal full-length CFTR mRNA in nasal epithelium was sufficient to ameliorate pulmonary disease severity relative to F508del homozygotes [7], and CFTR modulator therapy shows that partial pharmacological restoration of CFTR function produces measurable clinical benefit [8]. The 5–8% figure describes the aggregate level of CFTR activity across an epithelium, whereas the values reported here describe the proportion of cells carrying full CFTR function in a mosaic epithelium; a tissue in which 6% of cells have normal function is not physiologically equivalent to one in which every cell has 6% of normal function. The numerical proximity between this minimum detectable fraction and these benchmarks is coincidental, and we draw no inference from it. The implication of our data is narrower and concerns assay interpretation: a negative or modest FIS result after gene editing should not be read as an absence of correction unless the corrected fraction exceeds the limits established here.

These considerations place the present work within a broader effort to establish organoid-based functional assays as decision-support tools. In CF, analytical validation of the FIS assay is well advanced for intestinal organoids: a four-laboratory European study reported high intra-assay and within-subject repeatability and high between-laboratory reproducibility over two years, with 78–100% agreement in classifying modulator responses when predefined thresholds were applied [16], building on a standardized protocol with defined reference organoid lines [17]. No equivalent framework yet exists for airway or iPSC-derived models, which is one reason we regard the percentages reported here as properties of this model rather than transferable constants and why we recommend that 0% and 100% reference conditions be included in every run—an approach analogous to the “floating cut-off” strategy proposed for intestinal organoids where residual between-laboratory variability cannot be eliminated [16]. The step beyond analytical validation is less settled: among F508del homozygotes, FIS indicated responsiveness to CFTR modulators but did not discriminate high from low clinical responders, and higher swelling did not predict better clinical outcomes [18]. This is a useful corrective measure, and it is consistent with our own position that the thresholds reported here describe what the assay can resolve rather than what a patient would gain. Comparable questions of standardization, quality control, and the passage from an ex vivo readout to a clinical decision are being addressed in parallel for patient-derived tumor organoids in oncology [19].

Several limitations should be considered when interpreting these results. First, the chimeric iLO model is an in vitro system that lacks immune responses and native tissue architecture, both of which may alter the relationship between the proportion of corrected cells and functional outcome in vivo. Second, a single CFTR-wild-type iPSC line (Mak-f) was used, and only the F508del/F508del genotype was represented among CF cells. The values reported here should therefore be read as properties of this model system rather than as universal constants. The fraction of CFTR-competent cells required to reach a given area ratio scales inversely with the CFTR-dependent secretion contributed by each such cell, so wild-type lines differing in per-cell CFTR activity, or in differentiation efficiency and organoid cellular composition, would shift it; on the CF side, a residual-function genotype would raise the baseline of the CF-only condition and compress the dynamic range. The 6% value is additionally a statement about the precision of this dataset and would move with the number of wells and experiments contributing to the model, whereas the area ratio cutoffs are better anchored, since 1.21 had already been applied in unmodified iLOs [13], although it was among the thresholds evaluated here rather than an independent confirmation; it is their translation into a percentage of corrected cells that is model-specific. The percentages should therefore be re-derived for new lines against the 0% and 100% reference conditions recommended above, which were included in every run of this study. Third, the wild-type component of all chimeras was derived from a single lentivirally labeled Mak-f clone. The additional RT-qPCR measurements (Table S2) do not establish unchanged host-gene expression, because they compare a single-cell-derived clone with the polyclonal population from which it was isolated, so insertional effects, founder effects, and culture adaptation are confounded by construction. For *AP2A1*, the signal lay at the limit of detection in both lines (Ct above 38), and although the primer pair amplified efficiently from a positive-control cDNA, the comparison between the two lines is not quantitative, and no statement about this locus follows from it. For *LINC01118*, the transcript was less abundant in the clone by 2.14 cycles. Because the provirus occupies a single allele, complete silencing of that allele could lower a transcript by at most one cycle, so a decrease of this size is larger than disruption of the targeted allele alone could produce and argues against a simple cis-effect without excluding one, as allele-biased expression at this locus was not assessed. This ceiling argument applies only to reduced expression and does not extend to *SCAI*, which was 7.7-fold more abundant in the clone; an increase of that kind cannot arise from disruption of the targeted allele, and a trans-effect of the integration and the clonal and culture history of the subline remain equally compatible with it and are confounded in the present comparison. The RT-qPCR data are therefore not definitive either for or against an integration-related effect on expression at these loci. Resolving the contribution of individual integrations would require several independent GFP^+^ clones carrying different integration sites, or non-transduced subclones of the same parental line. Such effects could influence the observed FIS calibration. The reported relationship therefore characterizes the present labeled-cell model, and the estimated wild-type fractions should not be assumed to transfer unchanged to other clones or labeling strategies. Measurements of selected transcript regions also do not establish full transcript integrity or exclude vector–cellular chimeric transcripts [20,21]. Fourth, the ROC analysis treats wells as independent observations, although wells within an experiment share a cell suspension, a plate, and a batch of basement-membrane matrix; the sensitivity and specificity reported for the 1.16 and 1.21 cutoffs are therefore likely to be optimistic, and the mixed-effects analysis, which models this clustering explicitly, provides the more conservative estimate. Fifth, the wild-type fraction was quantified as the percentage of GFP-positive cells. Transcriptional silencing of the lentiviral GFP cassette during differentiation or organoid culture would cause CFTR-competent Mak-f cells to be scored as GFP-negative and would therefore underestimate the true wild-type fraction, shifting the percentages reported here upward; GFP fluorescence remained stable over months of culture in the undifferentiated clone, but silencing in differentiated progeny was not assessed by a method independent of GFP itself.

In summary, this study establishes a chimeric iPSC-derived lung organoid model that enables a systematic, dose-dependent calibration of FIS assay sensitivity against defined fractions of CFTR-competent cells. The data demonstrate that the FIS response becomes statistically detectable from approximately 6% CFTR-competent cells when all experiments are analyzed jointly, whereas the fitted mean response reaches the experimental FIS cutoffs of 1.16 (80% sensitivity, 80% specificity) and 1.21 (98% specificity, 70% sensitivity) at approximately 14% and 34% CFTR-competent cells, respectively. The chimeric iLO model provides a practical framework for directly correlating cell fraction with functional outcome and may serve as a versatile platform for evaluating gene editing strategies, cell-based therapies, and their combinations with CFTR modulators—in particular, for assessing whether pharmacological potentiation can lower the threshold of corrected cells required to produce a detectable FIS response.

## 4. Materials and Methods

### 4.1. Cell Culture

The study was approved by the Ethics Committee of the Research Centre for Medical Genetics (Moscow, Russia) and conducted in accordance with the provisions of the Declaration of Helsinki (1964, as revised in 2013). Patients and healthy donors signed informed written consent forms as anonymous participants in the study and donors of biological materials.

HEK293T cells were maintained in growth medium consisting of DMEM (PanEco, Gorki Leninskiye, Russia) with 4.5 g/L glucose, 10% fetal bovine serum (FBS) (Biowest, Nuaillé, France), 1% GlutaMAX (Gibco, Grand Island, NY, USA), and 50 U/mL penicillin/streptomycin (PanEco, Gorki Leninskiye, Russia). Cells were cultured on standard tissue culture plates or flasks without any special coating. Passaging was performed when cells reached approximately 70% confluency. The cells were washed twice with Dulbecco’s phosphate-buffered saline (DPBS) without Ca^2+^/Mg^2+^, dissociated using 0.05% trypsin–EDTA (PanEco, Gorki Leninskiye, Russia), and incubated at 37 °C for 3–5 min. Trypsin activity was neutralized by adding complete growth medium. The cell suspension was then centrifuged at 400× *g* for 5 min. The pellet was resuspended in fresh medium, and cells were subcultured at a split ratio of 1:5 to 1:7. The medium was changed every 2–3 days. Cells were cultured at 37 °C in a humidified atmosphere with 5% CO_2_.

The iPSC cultures were sourced from the “Russian Collection of Biological Samples of Hereditary Diseases” core facility (Research Center for Medical Genetics). iPSCs were maintained in TeSR™-E8™ (STEMCELL Technologies, Vancouver, BC, Canada) on a culture dish coated with Matrigel (Corning, Corning, NY, USA). Three iPSC lines were used in the study: two lines from patients with CF carrying a homozygous F508del mutation in the CFTR gene (P1L5 and P7L2 cell lines) and one line from a donor with a WT/WT genotype in the CFTR gene (Mak-f cell line) [22,23].

### 4.2. Lentiviral Vector Production

Lentiviral particles encoding the target transgene were produced in HEK293T cells via calcium–phosphate transfection. One day prior to transfection, cells were seeded at 7 × 10^6^ cells per 10 cm dish pre-coated with gelatin and maintained in antibiotic-free growth medium. Transfection reagents, including HBS buffer (pH 7.05–7.1) and 2 M CaCl_2_ solution, were prepared in advance for the entire experiment; HBS aliquots were stored at −20 °C and CaCl_2_ at 4 °C. Two to three hours before transfection, the medium was replaced with DMEM. For each sample, a transfection mixture was prepared by combining 75 µL of 2 M CaCl_2_, plasmid DNA, and nuclease-free water to a final volume of 750 µL. The solution was added dropwise to 750 µL of HBS buffer with continuous gentle mixing. The total plasmid DNA per dish was 18 µg, comprising an equimolar ratio of the packaging plasmid psPAX2 (psPAX2 was a gift from Didier Trono (Addgene plasmid #12260)), the envelope plasmid pVSV-G (pVSV-G was a gift from Akitsu Hotta (Addgene plasmid #138479)), and the transfer plasmid pULTRA (pULTRA was a gift from Malcolm Moore (Addgene plasmid #24129)). The combined solution was incubated at room temperature for 10–15 min to allow precipitate formation before being added dropwise to the cells. Following a 5.5-h incubation, the transfection medium was aspirated and replaced with fresh antibiotic-free growth medium. Viral supernatant was harvested 72 h post-transfection, clarified by centrifugation at 300 *g* for 5 min, and sterilized by filtration through a 0.45 µm membrane. The resulting viral preparation was aliquoted and stored at −70 °C.

### 4.3. Lentiviral Transduction

To enable tracking and quantification in mixed cultures, iPSC line Mak-f was transduced with a lentiviral vector encoding GFP (pULTRA-GFP). iPSC line Mak-f was cultured in a 24-well plate, and upon reaching approximately 60–70% confluence (4.0 × 10^5^ cells per well), the medium was completely replaced with 700 μL of fresh TeSR-E8 supplemented with a ROCK inhibitor (STEMCELL Technologies, Canada). The viral mixture containing 300 μL of the lentiviral vector was then added to each well. After 24 h of exposure to the vector in TeSR-E8, the inoculum was removed, and the cells were cultured for an additional 3–4 days before expansion. GFP-positive cells were enriched by FACS using a FACSAria III (BD Biosciences, Franklin Lakes, NJ, USA), yielding highly GFP-expressing populations that were then subjected to limiting dilution into 96-well plates for clonal expansion. Transduction efficiency was determined by flow cytometry before cloning, as the percentage of GFP-positive cells in the bulk transduced population, with untransduced Mak-f cells used to set the GFP-negative gate, reached 97%.

### 4.4. Lentiviral Integration Site Analysis

To identify the genomic integration loci of the lentiviral vector in the Mak-f-iPSC-GFP cell line, we performed targeted enrichment of genomic DNA fragments containing proviral insertions followed by high-throughput sequencing. The enrichment system was designed based on the sequence of the pULTRA plasmid used for lentiviral production. Custom biotinylated RNA oligonucleotide probes were synthesized to capture genomic DNA fragments complementary to the vector’s LTRs.

DNA libraries were prepared using the IGT^®^ Enzyme Plus Library Prep Kit V3 (Cat# C11112, iGeneTech Bioscience Co., Beijing, China) according to the manufacturer’s protocol. For ligation, the IGT^®^ Adapter & UDI Primer 1-96 set (Cat# C10042, iGeneTech Bioscience Co., China) was used. Intermediate purification steps were performed with IGT^®^ Pure Beads (Cat# C80661, iGeneTech Bioscience Co., China). Enrichment of adapter-ligated target fragments was carried out using the TargetSeq One^®^ Hyb & Wash Kit v3.0 (Cat# C11614, iGeneTech Bioscience Co., China) following the supplier’s instructions. The resulting libraries were sequenced on GenoLab M (Genemind, Shenzhen, China) with paired-end 150 bp reads. The obtained reads were mapped to the modified human reference genome (assembly hg38) with the additional chromosome identical to the lentiviral insert. Integration loci were identified as unique positions at the LTR-genome junction based on the presence of chimeric reads containing both vector and flanking genomic DNA sequences.

Transcript levels at the three intronic integration sites were quantified by RT-qPCR in the GFP^+^ clone and the parental Mak-f line; primer sequences are provided in Table S1. Reactions were run in technical triplicate, and relative transcript levels were calculated as FC = 2 − ΔΔCt, with B2M as the reference gene, taking ΔCt = Ct(target) − Ct(B2M) separately in each line and ΔΔCt = ΔCt(GFP^+^ clone) − ΔCt(parental line). Primer sequences (Table S1), reaction conditions, the positive and no-template controls, and the full normalization scheme are given in the Supplementary Materials.

### 4.5. Characterization of a GFP-Positive iPSC Clone

A GFP-positive Mak f iPSC clone was characterized by immunofluorescence analysis of pluripotency markers and by its ability to form embryoid bodies (EBs), followed by spontaneous differentiation into the three germ layers (mesoderm, ectoderm, and endoderm).

For immunofluorescence analysis of pluripotency markers, GFP-positive Mak f clone was fixed in cold 4% formalin for 15 min at room temperature (RT, 25 °C). Cells were washed twice with DPBS, permeabilized in 0.1% Tween 20 for 10 min at RT, washed three times with DPBS, and then blocked in 1% BSA (Paneco, Russia) containing 0.1% Tween 20 for 30 min at RT. Primary antibodies against OCT4 (cat. no. ab18976, Abcam, Cambridge, UK), SSEA4 (cat. no. 414000, Thermo Fisher Scientific, Waltham, MA, USA), SOX2 (cat. no. 79351, Thermo Fisher Scientific, USA), and TRA 1 60 (cat. no. 41 1000, Thermo Fisher Scientific, Waltham, MA, USA) were then added, and cultures were incubated for 1 h at RT, followed by three DPBS washes. Subsequently, secondary antibodies Goat Anti-Mouse IgG H&L (Alexa Fluor 488, RRID AB_2630356, Abcam, UK) and Goat Anti-Rabbit IgG H&L (Alexa Fluor 594, RRID AB_2650602, Abcam, UK) were applied for 1 h at RT. After three additional washes with DPBS, nuclei were counterstained with DAPI (Abcam, UK) for 10 min at RT. Stained cells were imaged using an automated Lionheart FX imaging system (BioTek, Winooski, VT, USA).

EB formation was performed as described previously [22]. Briefly, the GFP-positive Mak f clone was detached from the culture surface and transferred to 6-well plates pre-coated with poly-L-lysine to reduce cell adhesion and cultured in TeSR-E8 medium. By day 2, spheroid formation was observed. Spheroids were cultured for one month with medium changes every 3 days. The first medium change was performed on day 4 by replacing the medium with a 1:1 mixture of TeSR-E8 and ES medium. ES medium consisted of Advanced DMEM/F12 (Gibco), 20% KnockOut Serum Replacement (Gibco), GlutaMAX (Gibco), 1% non-essential amino acids (Gibco), and 100 U/mL penicillin plus 100 µg/ml streptomycin (PanEco, Russia). The second medium change was performed on day 7, replacing the medium with EB medium composed of a 1:1 mixture of ES medium and fibroblast medium without FBS. Fibroblast medium without FBS consisted of DMEM/F12 (Gibco), GlutaMAX (Gibco), 1% non-essential amino acids (Gibco), and 100 U/mL penicillin plus 100 µg/ml streptomycin. For the next 14 days, half of the medium volume was replaced with EB medium every 2–3 days, with a gradual increase in fetal bovine serum (FBS) concentration from 1% to 10%. Mature EBs were then transferred to high-adhesion culture dishes coated with 0.1% gelatin to allow attachment and outgrowth of cells and were maintained for an additional 14 days in EB medium supplemented with 10% FBS. Subsequently, immunofluorescent staining for markers of the three germ layers (ectoderm, endoderm, and mesoderm) was performed using the same protocol as described above. The following primary antibodies were used: vimentin (cat. no. ab92547, Abcam, UK), βIII tubulin (cat. no. ab78078, Abcam, UK), and α-fetoprotein (cat. no. ab3980, Abcam, UK). Cell cultures during maintenance and after immunofluorescent staining were imaged using the Lionheart FX system.

Proliferation of the GFP-positive clone and of the untransduced parental Mak-f population was compared by live phase-contrast imaging on the same system with automated nuclear counting at 0 and 96 h, three wells per group. Population doublings were calculated as PD = log_2_(N_96_/N_0_), and population doubling time as PDT = Δt · ln2/ln(N_96_/N_0_); the well was the unit of replication; groups were compared by two-sided Welch’s *t*-test, and the two timepoints within a group by paired *t*-test on log_2_ counts. Imaging settings, segmentation, and the assumptions of this analysis are given in the Supplementary Materials (Figure S2).

### 4.6. Karyotyping

A GFP-positive Mak-f iPSC clone at the 5th passage and approximately 70% confluence was arrested by 0.1 μg/mL demecolcine (Sigma-Aldrich, St. Louis, MO, USA), harvested by trypsinization, hypotonized for 13 min in 0.075 M KCl at 37 °C, and fixed using standard cytogenetic procedures. Slides were stained with Vectashield mounting medium with DAPI (Vector Laboratories, Newark, CA, USA) contrasted with 0.3 mg/mL Actinomycin D (Serva, Heidelberg, Germany). At least 15–20 metaphase images were analyzed according to ISCN 2024 nomenclature.

### 4.7. Derivation of Induced Airway Basal Cells

A GFP-positive Mak-f iPSC clone was differentiated into induced airway basal cells (iBCs) using a previously published protocol [12]. Briefly, hiPSCs were sequentially differentiated into definitive endoderm (activin A, CHIR99021), anterior foregut endoderm (SB431542, dorsomorphin), and NKX2-1^+^ lung progenitors (CHIR99021, BMP4, retinoic acid). At the lung progenitor stage, cells were cultured in PneumaCult-Ex Plus medium (STEMCELL Technologies) supplemented with 1 µM A83-01 (Tocris), 1 µM DMH1 (Tocris), 1 µM hydrocortisone (Sigma-Aldrich), 50 U/mL penicillin, and 50 µg/mL streptomycin to promote acquisition of an airway basal cell phenotype. After expansion, NGFR^+^ (CD271^+^) airway basal-like cells were enriched by flow cytometry as described previously and maintained in PneumaCult-Ex Plus under the same supplement conditions.

To confirm successful differentiation, immunofluorescent staining for airway basal cell markers was performed as described above. Primary antibodies against TP63 (cat. no. 703809, Thermo Fisher Scientific, USA) and KRT5 (cat. no. ab52635, Abcam, UK) were used. Stained cells were imaged using a Lionheart FX imaging system. Two additional iPSC-derived iBC lines (P1L5 and P7L2) had been generated and characterized previously [12].

### 4.8. Generation of Chimeric Induced Lung Organoids

Chimeric iLOs were generated from GFP-positive iBCs Mak-f (WT/WT in CFTR) and GFP-negative iBCs P1L5 or P7L2 (F508del/F508del in CFTR) according to a previously described protocol [12]. Briefly, iBCs were detached from culture plates using Versene (Paneco, Russia), and viable cells were counted with an automated cell counter (RWD, Shenzhen, China). A total of 1 × 10^3^ cells were transferred into a microcentrifuge tube and centrifuged at 2500 rpm for 5 min. The cell pellet was resuspended in Matrigel, and 10 µL droplets were plated into wells of a 48-well plate. The droplets were incubated for 40 min in a CO_2_ incubator, followed by the addition of the lung organoid culture medium. The lung organoid medium consisted of 75% IMDM (Thermo Fisher Scientific, USA), 25% Ham’s F12 (PanEco, Russia), 100× B-27 (Thermo Fisher Scientific, USA), 200× N2 (PanEco, Russia), 0.05% bovine serum albumin solution (Sigma-Aldrich, USA), 0.45 mM 1-thioglycerol (Sigma-Aldrich, USA), 100× GlutaMAX (Thermo Fisher Scientific, USA), 0.05 mg/mL L-ascorbic acid (Sigma-Aldrich, USA), 100× penicillin–streptomycin (PanEco, Russia), 10 ng/mL FGF7 (R&D Systems, Minneapolis, MN, USA), 100 ng/mL FGF10 (R&D Systems, USA), 10 ng/mL EGF (R&D Systems, USA), and 3 µM CHIR99021. Seven experimental groups of iLOs were assembled with the planned ratios of cells as follows: (1) P1L5/P7L2—100%, Mak-f—0%; (2) P1L5/P7L2—99%, Mak-f—1%; (3) P1L5/P7L2—95%, Mak-f—5%; (4) P1L5/P7L2—90%, Mak-f—10%; (5) P1L5/P7L2—85%, Mak-f—15%; (6) P1L5/P7L2—80%, Mak-f—20%; and (7) P1L5/P7L2—0%, Mak-f—100%.

### 4.9. Forskolin-Induced Swelling Assay

To perform the FIS assay, on day 7 of chimeric iLO formation, organoids were passaged into a 96-well plate with a 3 μL drop volume in lung organoid medium. On the first day of analysis, forskolin at a final concentration of 10 μM was added to the wells containing the organoids, and then images were acquired on a Lionheart FX automated imager in the BF channel with a 4× magnification as described in the section above. The time-course of swelling over the 24 h window, performed on a separate plate with imaging every 2 h, is described in the Supplementary Materials. Then, after incubation for 24 h, images were acquired on a Lionheart FX automated imager again. The results were analyzed using a semi-automated algorithm published previously [24]. Briefly, organoid segmentation was performed using a trained U-Net convolutional neural network followed by CellProfiler post-processing for instance segmentation, artifact removal, and organoid tracking across timepoints. The cross-sectional area of each segmented organoid was extracted from binary masks, and the area ratio was calculated as the total 2D projected organoid area at t = 24 h divided by the corresponding area at t = 0 h for the same organoid. Group-level statistical comparisons were performed in GraphPad Prism (version 9.1.1).

### 4.10. Flow Cytometry Assay

Flow cytometry was performed on chimeric iLOs immediately after the FIS assay. For this, organoids were collected and dissociated using Trypsin-EDTA solution (Paneco, Russia) for 15 min, centrifuged at 7700 rpm for 1 min, and resuspended in DPBS without Ca^2+^/Mg^2+^. The cells were then stained with Hoechst 33342 (for nuclear visualization). Prior to flow cytometry, propidium iodide (to identify dead cells) was added to the samples, and the cell suspension was passed through a 50 µm filter. Flow cytometric analysis was performed using a CytoFLEX S flow cytometer (Beckman Coulter, Indianapolis, IN, USA). Debris and aggregates were excluded on forward and side scatter; Hoechst 33342-positive events were retained as nucleated cells, and propidium iodide-positive events were excluded as non-viable. The GFP gate was set on dissociated organoids of the 0% Mak-f condition of the same experiment, which contain no GFP-labeled cells, and the actual percentage of Mak-f cells in a condition was taken as the percentage of GFP-positive events among viable nucleated single cells.

### 4.11. Statistical Data Analysis

The statistical analysis and representation of the data were conducted using GraphPad Prism (version 9.1.1) and R (version 4.3.3) with lme4 (version 1.1.35.1), lmerTest (version 3.1.3), pbkrtest (version 0.5.2), emmeans (version 1.10.0) and splines (base R). FIS was quantified as the organoid area ratio, and each well was summarized by the geometric mean of the area ratios of all organoids it contained (479 wells, 42 conditions, 6 independent experiments, 2 CF patient-derived lines). The actual percentage of GFP-positive Mak-f cells was measured once per condition, because all wells belonging to a given condition were seeded from a single cell suspension at a defined Mak-f-to-CF cell ratio and were pooled prior to dissociation. Well-level geometric means were log-transformed and analyzed with a linear mixed-effects model fitted by REML, as log(area ratio) ~ line + ns(sqrt(GFP-positive %), df = 3) + (1 | experiment) + (1 | experiment–condition), where experiment denotes one independent experiment on one plate and condition denotes one cell suspension, that is, one flow cytometry-measured GFP-positive percentage within that experiment, distributed over several wells. Fixed effects were the CF line and the actual percentage of GFP-positive Mak-f cells, entered as a natural cubic spline with three degrees of freedom on the square-root scale. Fixed effects were tested by F tests with Kenward–Roger denominator degrees of freedom, all tests being two-sided. Residuals were homoscedastic and the random effects approximately normal; the residual distribution was symmetric with mildly heavy tails. Estimated marginal means, averaged over the two CF lines, were computed over the 0–100% range and contrasted with the estimate at 0%. A 95% simultaneous confidence band for these differences was constructed using the multivariate-t distribution, which accounts for the correlation between neighboring points of the dose range; the detection threshold was taken as the smallest fraction at which the band excluded the CF-only value. Contrasts are reported back-transformed from the log scale as fold changes with 95% CIs. To determine the experimental FIS cutoffs, ROC analysis was performed utilizing Youden’s J statistic and custom thresholds. Wells of the 0% Mak-f conditions were treated as negative cases and all wells of the chimeric and 100% Mak-f conditions as positive cases; the unit of analysis was the well-level geometric mean of organoid area ratios. To estimate the percentage of GFP-positive cells corresponding to these calculated cutoffs, swelling data from both patient lines were averaged across technical replicates for each group. A four-parameter logistic (4PL) sigmoidal model was then fitted to the aggregated data, allowing the exact GFP-positive cell percentages to be calculated from the swelling thresholds via inverse interpolation.

## Data Availability

The data presented in this study are available upon request from the corresponding author.

## Supplementary Materials

The following supporting information can be downloaded at https://www.mdpi.com/article/10.3390/ijms27219765/s1.

## Abbreviations

The following abbreviations are used in this manuscript:

4PL	Four-parameter logistic (model)
BSA	Bovine serum albumin
CF	Cystic fibrosis
CFTR	Cystic fibrosis transmembrane conductance regulator
CI	Confidence interval
DPBS	Dulbecco’s phosphate-buffered saline
EB	Embryoid body
ER	Endoplasmic reticulum
LV	Lentiviral vector
RT-qPCR	Reverse transcription quantitative polymerase chain reaction
SD	Standard deviation
FACS	Fluorescence-activated cell sorting
FBS	Fetal bovine serum
FEV1	Forced expiratory volume in 1 s
FIS	Forskolin-induced swelling
GFP	Green fluorescent protein
iBC	Induced airway basal cell
IL-8	Interleukin 8
iLO	Induced lung organoid
iPSC	Induced pluripotent stem cell
ISCN	International System for Human Cytogenomic Nomenclature
LTR	Long terminal repeat
mRNA	Messenger RNA
ROC	Receiver operating characteristic
WT	Wild-type
