# Supplementary material for: Chimeric iPSC-Derived Lung Organoids Reveal the Minimum Wild-Type Cell Fraction Detectable by the Forskolin-Induced Swelling Assay in Cystic Fibrosis
Source: Int J Mol Sci. 2026 Oct 30;27(21):9765. doi: 10.3390/ijms27219765 (PMC13763470; doi:10.3390/ijms27219765)
Supplement: Supplementary file 1 [file ijms-27-09765-s001.zip › ijms-4538557-supplementary.pdf]

Supplementary material

RNA isolation, reverse transcription and RT-qPCR

Material and methods

Total RNA was isolated from the GFP+ Mak-f monoclonal line and from the untransduced parental Mak-f line using Column total RNA isolation kit (cat. n. RU-250, Biolabmix, Russia) according to the manufacturer's protocol, with on-column DNase I treatment. RNA concentration and purity were assessed on NanoDrop spectrophotometer (Thermo Fisher Scientific, USA). Reverse transcription was carried out on 500 ng of total RNA per reaction using MMLV RT kit (cat. n. SK021, Evrogen, Russia) with oligo-dT priming. 5 µL of the resulting cDNA was used per qPCR reaction.

Expression of *AP2A1*, *SCAI* and *LINC01118* was assessed by qPCR using primer pairs designed so that at least one primer of each pair spanned an exon–exon junction of the corresponding spliced mRNA while the other lay within an adjacent exon, which restricts amplification to cDNA derived from processed mRNA. Because the junction-spanning design precludes amplification from genomic DNA, no-reverse-transcriptase controls were not included. Primer design was constrained to amplicons no longer than 250 bp. Following preliminary evaluation of amplification performance, the primer pairs listed in Table S1 were selected.

Table S1. Primer pairs used for RT-qPCR

Forward primer	Forward primer sequence	Reverse primer	Reverse primer sequence	Amplicon
AP2A1_E2.3F	GCTCCAAGTTCAAAGGAGACAA	AP2A1_E3R	GTCATGGCCAAGCAGGAAGA	92 bp
SCAI_E18F	CATCCCTTGGCAGTTGTGGA	SCAI_E16.17R	CATTCACGTTCAATAGATCAGGCA	163 bp
LINC01118_F	GTCACAGCTTTGAGAGGCAA	LINC01118_R2	AGCCAAGTAGGGCAGTCAGAA	125 bp
B2M-192_F	GCTCGCGCTACTCTCTCTTT	B2M-192_R	TCTGAATGCTCCACTTTTTCAA	192 bp

An annealing temperature of 60 °C was used for all primer pairs. Real-time PCR was performed with Taq DNA polymerase (5 U/µL; Evrogen, Russia) at a final concentration of 0.04 U/µL in a total reaction volume of 25 µL, with the proprietary Taq Turbo buffer (Evrogen, Russia), 0.4 mM dNTPs, 0.2 µM of each primer and 1× SYBR Green I. Amplification comprised an initial denaturation at 95 °C for 3 min followed by 40 cycles of 95 °C for 20 s, 60 °C for 15 s and 72 °C for 15 s, with fluorescence acquired during the extension step of each cycle. For *SCAI*, an additional acquisition step at 80.5 °C for 15 s was included to exclude signal from primer dimers. Amplification specificity was assessed by melting curve analysis from 65 °C to 95 °C in 0.5 °C

increments. All reactions were run in technical triplicate with no-template controls (NTC) in each run.

Amplification of the housekeeping gene *B2M* confirmed the presence of amplifiable cDNA in each reaction. *AP2A1/SCAI* and *LINC01118* were analysed in separate qPCR runs. For each target, the parental and GFP-positive samples and the corresponding *B2M* reference reactions were included in the same run. Total RNA-derived cDNA from an in-house myoblast cell line was included as a positive control for *AP2A1* primer pairs. *B2M* was also used as the reference gene for normalization and was measured alongside the targets in each run. Relative transcript levels in the GFP+ clone compared with the parental line were calculated as FC = 2−ΔΔCt, with ΔCt = Ct(target) − Ct(*B2M*) determined separately in each line and ΔΔCt = ΔCt(s2) − ΔCt(s1).

Results

To assess whether proviral insertion had perturbed transcription at these loci, steady-state transcript levels of *AP2A1*, *SCAI* and *LINC01118* were measured by RT-qPCR in the GFP^+ clone and in the untransduced parental Mak-f line (Table S2). *SCAI* showed no reduction in the GFP^+ clone and was more abundant there (ΔΔCt −2.95 ± 1.02, 7.7-fold). *AP2A1* amplified at the detection limit in both lines (mean Ct 38.2 and 39.3), so the comparison between them is not informative (ΔΔCt −0.29 ± 1.16, 1.2-fold); the same primer pair amplified the myoblast positive control at Ct 25.6, which is evidence of primer function only and does not make the comparison between the two lines quantitative. *LINC01118* was less abundant in the clone (ΔΔCt +2.14 ± 0.40, 4.4-fold). Because the provirus occupies a single allele at each locus, complete silencing of that allele could reduce a transcript by at most one cycle; the difference observed for *LINC01118* is twice that ceiling and argues against a cis-effect without excluding one, while differences at or below the ceiling are not resolvable in a comparison between a single clone and its polyclonal parent. This ceiling argument applies only to reduced expression and does not extend to *SCAI*, which was 7.7-fold more abundant in the clone: an increase of that kind cannot arise from disruption of the targeted allele, whereas a trans-effect of the integration and the clonal and culture history of the subline remain equally compatible with it and are confounded in the present comparison. Because the GFP^+ clone is a single-cell-derived subline of the polyclonal parental population, insertional effects, founder effects and culture adaptation cannot be separated in this design, and the RT-qPCR data are therefore not definitive either for or against an integration-related effect on expression at these loci.

Table S2. RT-qPCR quantification of transcripts at the three intronic lentiviral integration loci. Mean Ct ± SD of technical replicates is given for the untransduced parental Mak-f line and the GFP^+ Mak-f monoclonal line (n = 3)

**Target**	**Line**	**Mean Ct ± SD**	**ΔCt vs B2M**	**ΔΔCt**	**Fold change**
Run 1 (*AP2A1, SCAI*)					

AP2A1	Mak-f parental	38.23 ± 0.97	+10.55	—	—
AP2A1	Mak-f-GFP	39.34 ± 0.35	+10.26	−0.29 ± 1.16	1.2× higher
SCAI	Mak-f parental	34.73 ± 0.52	+7.06	—	—
SCAI	Mak-f-GFP	33.18 ± 0.69	+4.11	−2.95 ± 1.02	7.7× higher
B2M	Mak-f parental	27.67 ± 0.34	—	—	—
B2M	Mak-f-GFP	29.07 ± 0.41	—	—	—
AP2A1	Myoblast control	25.65 ± 0.14	+0.91	—	—
B2M	Myoblast control	24.73 ± 0.66	—	—	—
Run 2 (*LINC01118*)					
LINC01118	Mak-f parental	28.53 ± 0.14	−10.84	—	—
LINC01118	Mak-f-GFP	25.67 ± 0.30	−8.71	+2.14 ± 0.40	4.4× lower
B2M	Mak-f parental	39.37 ± 0.23	—	—	—
B2M	Mak-f-GFP	34.37 ± 0.03	—	—	—
all targets	NTC	no amplification	—	—	—

Immunofluorescence analysis of airway basal cell identity and exclusion of alternative lineage markers

Material and methods

To confirm that the differentiated induced airway basal cells (iBCs) acquired a lung-specific phenotype and did not aberrantly express markers of alternative epithelial lineages, immunofluorescence staining was performed on the GFP^+ Mak-f iBC line. Cells were fixed in cold 4% formalin for 15 min at room temperature (RT, 25°C), washed twice with DPBS, and permeabilized in 0.1% Tween-20 for 10 min at RT. Non-specific binding was blocked with 1% BSA (Paneco, Russia) containing 0.1% Tween-20 for 30 min at RT. Primary antibodies (Table S3) were applied for 1 h at RT, followed by three washes with DPBS. Secondary antibodies — Goat Anti-Mouse IgG H&L (Alexa Fluor 488, RRID AB_2630356, Abcam, USA) and Goat Anti-Rabbit IgG H&L (Alexa Fluor 594, RRID AB_2650602, Abcam, USA) — were applied for 1 h at RT. After three additional DPBS washes, nuclei were counterstained with DAPI (Abcam, USA) for 10 min at RT. Stained cells were imaged using an automated Lionheart FX imaging system (BioTek, USA).

Table S3. Primary antibodies used for immunofluorescence analysis of iBCs

**Target**	**Antibody**	**Catalogue number**	**Manufacturer**
Airway basal cell markers	TP63	703809	Thermo Fisher Scientific, USA

Target	Antibody	Catalogue number	Manufacturer
	KRT5	ab52635	Abcam, USA
Intestinal progenitor cell markers	MUC2	ab134119	Abcam, USA
	LGR5	MA5-25644	Thermo Fisher Scientific, USA
Hepatic progenitor cell marker	AFP (α-fetoprotein)	ab169552	Abcam, USA

Results

Immunofluorescence staining confirmed that differentiated iBCs expressed the canonical airway basal cell markers TP63 and KRT5, consistent with successful acquisition of a lung epithelial progenitor phenotype. In contrast, no immunoreactivity was detected for markers of intestinal progenitor cells (MUC2 and LGR5) or hepatic progenitor cells (AFP), indicating that the differentiation protocol did not drive cells towards alternative epithelial lineages (Figure S1).

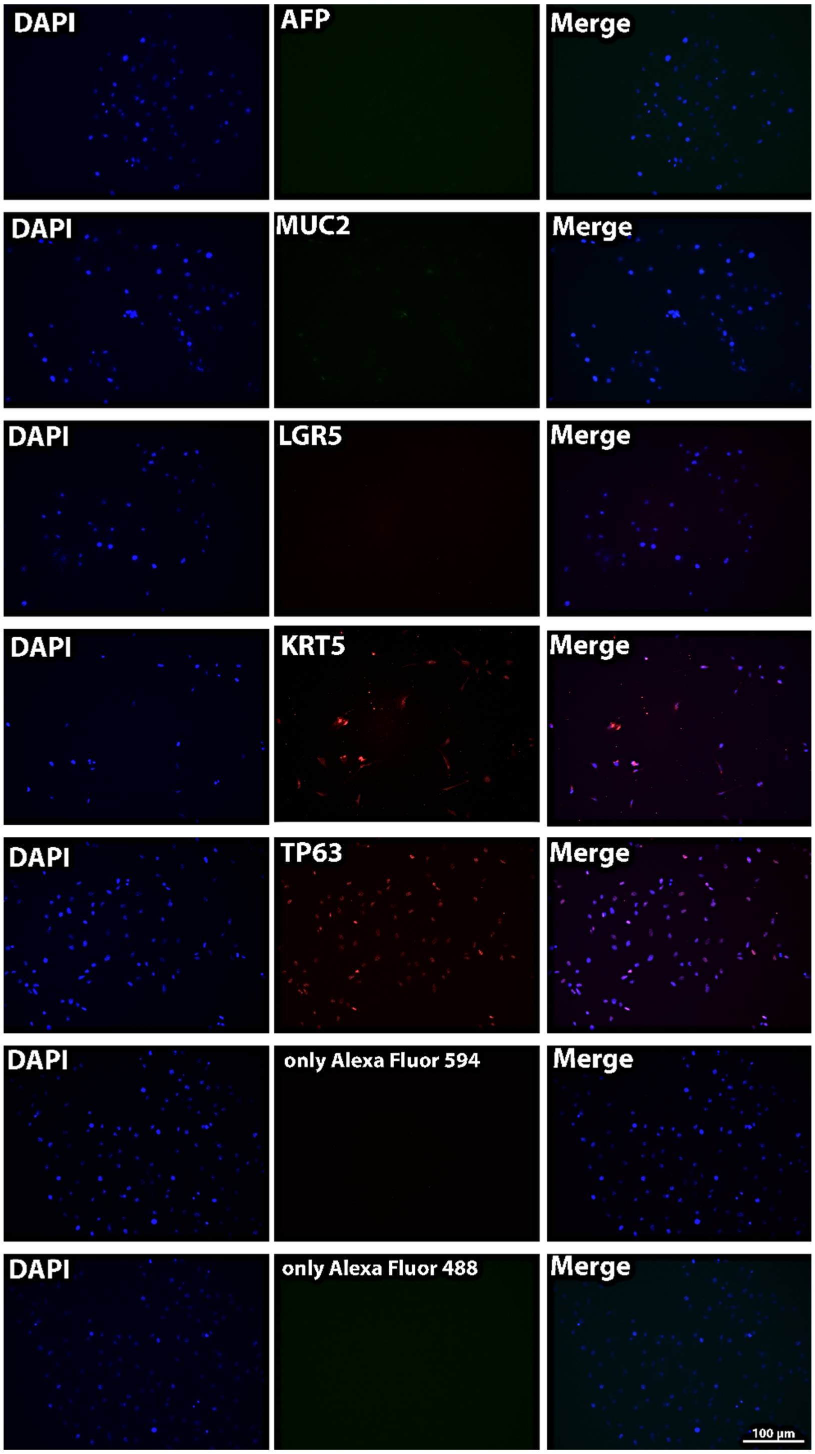

Figure S1. Immunofluorescence analysis confirming airway basal cell identity and absence of alternative lineage markers in differentiated iBCs (GFP^+ Mak-f iBC line). Cells stained positive for the airway basal cell markers TP63 and KRT5, and negative for intestinal progenitor markers MUC2 and LGR5, and hepatic progenitor marker AFP. Nuclei were counterstained with DAPI (blue). Scale bar, 100 µm

Proliferation assay

Material and methods

The untransduced parental Mak-f population and the GFP^+ Mak-f monoclonal subline were seeded in six-well plates at a nominally identical density (three wells per group) and imaged live on a Lionheart FX system (Agilent BioTek, USA) in phase contrast with a 10× objective (pixel size 0.6585 µm; field of view 0.4798 mm^2) at 0 and 96 h. Nuclei were segmented and counted automatically using a convolutional nucleus detector for transmitted-light images, excluding objects intersecting the frame border; cell number per field, surface density and confluence were recorded. Population doublings were calculated as $PD = \log_2(N_{96}/N_0)$ and population doubling time as $PDT = \Delta t \cdot \ln 2 / \ln(N_{96}/N_0)$, where N_0 and N_{96} are the cell numbers per field at 0 and 96 h. The well was taken as the unit of replication ($n = 3$ per group) and technical fields from the same well were averaged before analysis. Groups were compared by two-sided Welch's t-test and the two time points within a group by paired t-test on $\log_2$ counts; $\alpha = 0.05$. Analyses were performed in R 4.4.0. Because the number of cells present at 0 h differed between the groups, between-group comparisons of absolute cell number and surface density are confounded by this difference, whereas the normalized parameters PD and PDT are not; PDT additionally assumes exponential growth throughout the interval, an assumption that is not fully met in the GFP^+ group, which approached confluence by 96 h.

Results

To test whether clonal derivation of the GFP^+ subline had selected a more rapidly proliferating population, we compared the growth of GFP^+ Mak-f cells with that of the untransduced parental Mak-f population by live phase-contrast imaging with automated nuclear counting at 0 and 96 h. Both populations expanded significantly over the interval (paired t-test on $\log_2$ counts, $p = 0.004$ and $p = 0.002$ for GFP− and GFP^+ cells, respectively) and completed an indistinguishable number of population doublings (3.4 ± 0.4 versus 3.5 ± 0.3; $p = 0.643$), with indistinguishable population doubling times (28.8 ± 3.4 h versus 27.5 ± 2.1 h; $p = 0.621$). Growth trajectories of individual wells were parallel on a logarithmic scale (Figure S2). Lentiviral labelling and clonal expansion therefore did not confer a detectable proliferative advantage, although with three wells per group the comparison is powered to exclude only large differences.

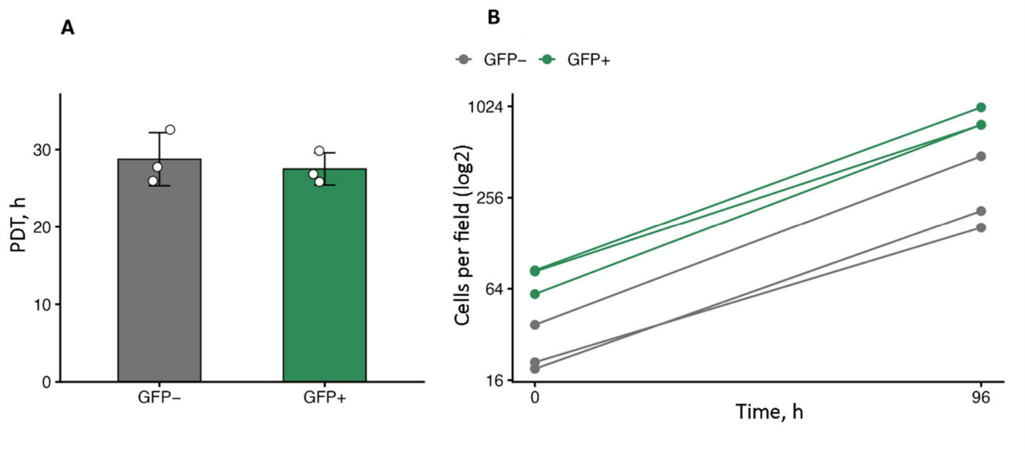

Figure S2**.** Population growth kinetics of the parental Mak-f population (GFP−) and the GFP^{+} Mak-f monoclonal subline. (A) Population doubling time (PDT); bars, mean; whiskers, ±SD; points, individual wells; the difference between groups was not significant ($p = 0.621$). (B) Cell number per well over time on a $\log_2$ scale; the parallel trajectories indicate comparable specific growth rates. $n = 3$ wells per group

Cellular composition of chimeric lung organoids

Material and methods

To characterize the cellular composition of chimeric iLOs and to visualize GFP-labelled wild-type cell distribution and *CFTR* expression patterns in chimeric and non-chimeric organoids, whole-organoid immunofluorescence staining was performed on two organoid groups: chimeric iLOs composed of 10% Mak-f WT/WT-GFP cells and 90% P1L5 F508del/F508del cells, and control iLOs composed exclusively of Mak-f WT/WT-GFP cells (100%). iLOs were collected from Matrigel droplets by mechanical dislodging on day 7 after formation and centrifuged for 5 s at 6300 × g. Organoids were fixed in chilled 4% formalin solution for 45 min at 4°C, permeabilized in cold 0.1% Tween-20 (Merck, Germany) for 10 min at 4°C, and centrifuged for 5 min at 70 × g at 4°C. The pellet was blocked with a cold solution of 0.1% Triton X-100 and 0.2% BSA in DPBS without Ca^{2+}/Mg^{2+} for 15 min at 4°C. Primary antibodies (Table S4) were then added and the suspension was incubated overnight at 4°C. Organoids were washed twice with a solution of 0.1% Triton X-100 and 0.2% BSA in DPBS for 2 h at 4°C, followed by addition of secondary antibodies Anti-Rabbit IgG H&L (Alexa Fluor® 647) (Abcam, cat. no. ab150075) or ABflo® 594-conjugated Goat Anti-Mouse IgG (H+L) (Abclonal, cat no. AS054) and incubation overnight at 4°C. After two additional washes (2 h each, 4°C), organoids were stained with DAPI (Abcam, cat. no. ab104139), then transferred onto glass slides, and covered with coverslips. Imaging was performed on a TCS SP8 confocal laser scanning microscope (Leica Microsystems, Germany).

Table S4. Primary antibodies used for immunofluorescence analysis of iLOs

Target	Antibody	Catalogue number	Manufacturer
Alveolar epithelial cells	AQP1	AF5231	Affinity Biosciences (China)
Alveolar epithelial cells	PDPN	DF12456	Affinity Biosciences (China)
Goblet cells	MUC5AC	DF9639	Affinity Biosciences (China)
Club cells	SCGB3A2	ab181853	Abcam, USA
CFTR-expressing cells	CFTR	ab13155	Abcam, USA
GFP-expressing cells	GFP	ab1218	Abcam, USA

Results

In chimeric iLOs composed of 10% Mak-f WT/WT-GFP and 90% P1L5 F508del/F508del cells, CFTR immunostaining revealed patchy CFTR-positive regions, consistent with a mosaic distribution of CFTR-expressing wild-type cells within a predominantly CFTR-deficient epithelium. In contrast, control iLOs composed of 100% Mak-f WT/WT-GFP cells exhibited widespread CFTR expression throughout the organoid epithelium. Organoids in both conditions contained AQP1- and PDPN-positive lung epithelial cells, as well as differentiated epithelial cells, including SCGB3A2-positive Club cells and MUC5AC-positive goblet cells, confirming that the organoids had acquired a structured, multicomponent epithelial architecture characteristic of mature lung organoids (Figure S3).

GFP immunostaining confirmed the presence of Mak-f WT/WT-GFP-derived cells within chimeric iLOs composed of 10% Mak-f WT/WT-GFP and 90% P1L5 F508del/F508del cells. In chimeric organoids, GFP-positive cells were distributed as discrete regions within the organoid epithelium, indicating successful incorporation of the wild-type cell fraction. In contrast, control organoids composed entirely of Mak-f WT/WT-GFP cells showed uniform GFP staining throughout the epithelial structure. The spatial distribution of GFP-positive cells in chimeric iLOs was consistent with the mosaic pattern of CFTR immunoreactivity observed in the same group (Figure S4).

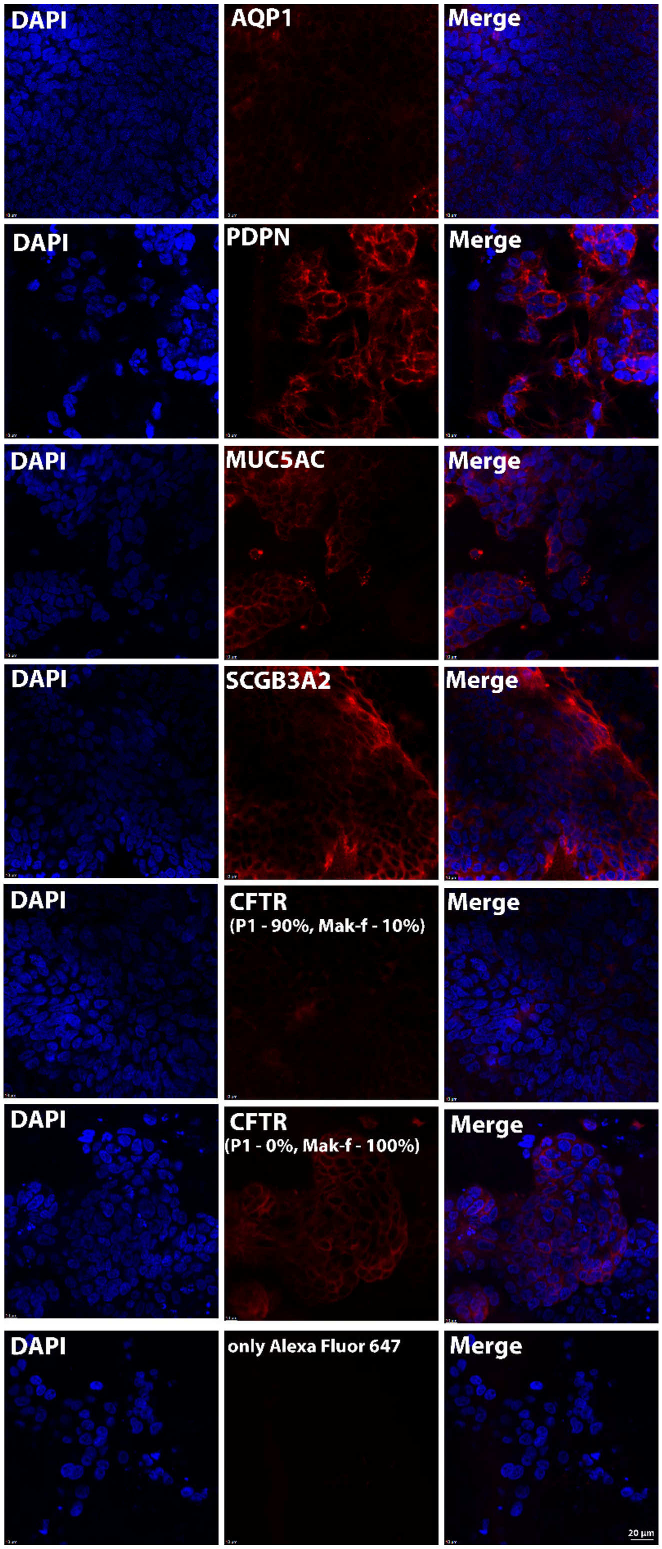

Figure S3. Cellular composition and CFTR distribution in chimeric and control iLOs. Representative confocal images of iLOs composed of 10% Mak-f WT/WT-GFP and 90% P1L5 F508del/F508del cells (10% indicates the planned seeding ratio; the actual fraction of GFP-positive cells was not determined by flow cytometry in this immunofluorescence-only experiment) and of control iLOs composed of 100% Mak-f WT/WT-GFP cells, stained for AQP1,

PDPN, MUC5AC, SCGB3A2 and CFTR; the bottom row is a secondary-antibody-only control. Nuclei were counterstained with DAPI (blue). Scale bar, 20 μm

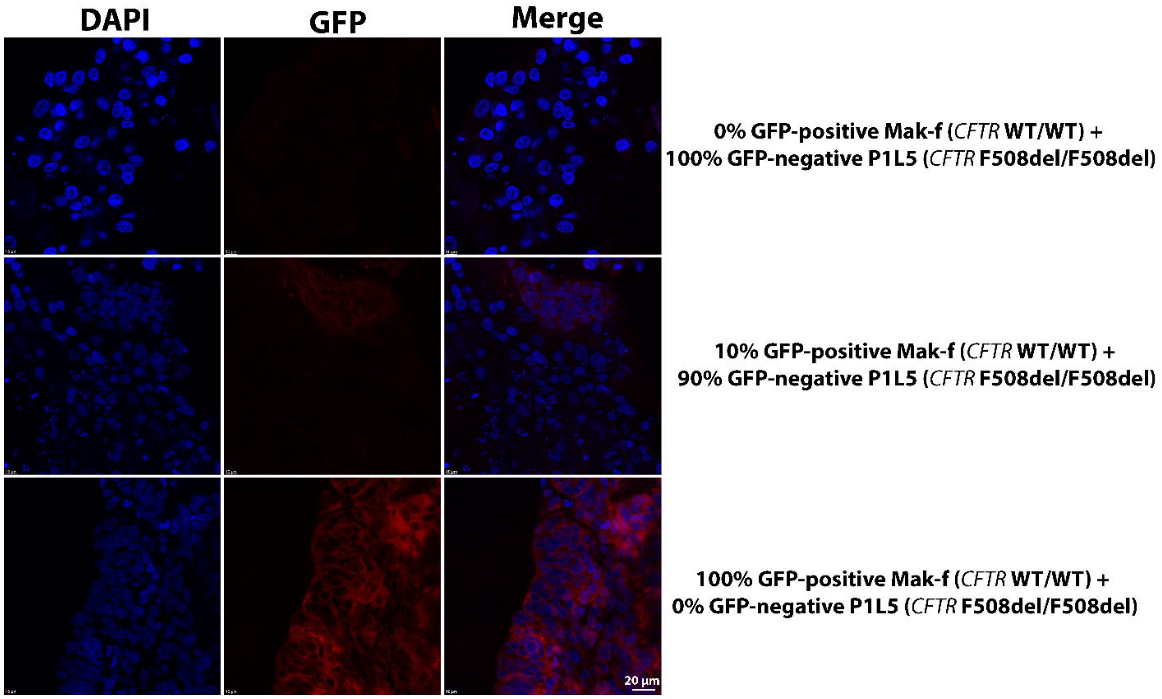

Figure S4. Distribution of GFP-labelled wild-type cells in chimeric and control iLOs. Representative confocal images of GFP immunostaining in iLOs assembled at planned ratios of 0%, 10% and 100% Mak-f WT/WT-GFP cells with the complementary proportion of P1L5 F508del/F508del cells; the 0% group contains no GFP-labelled cells and serves as the negative control. Nuclei were counterstained with DAPI (blue). Scale bar, 20 μm

Representative bright-field images of chimeric iLOs before and after forskolin stimulation

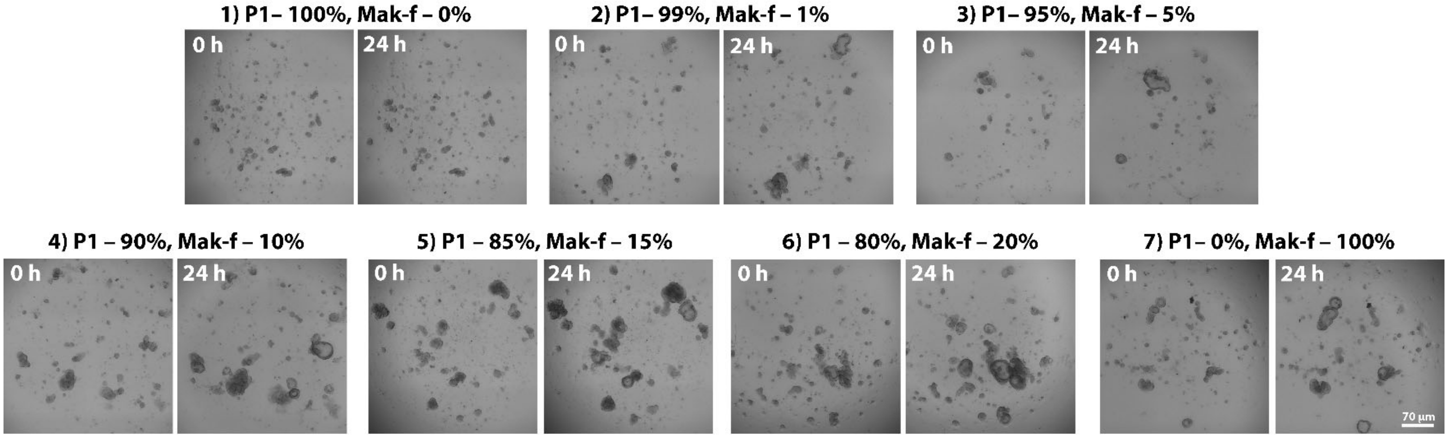

Figure S5. Representative bright-field images of chimeric iLOs (Mak-f WT/WT-GFP + P1L5 F508del/F508del) at t = 0 h and t = 24 h after forskolin addition. Images are shown for all seven experimental groups assembled at planned input ratios of 0%, 1%, 5%, 10%, 15%, 20%, and 100% Mak-f cells. Scale bar, 70 µm

Table S5. Cellular composition and area changes of chimeric lung organoids after 24 h of forskolin incubation. Values are condition means ± SD of well-level geometric means of per-organoid area ratios

**Group of chimeric iLOs**	**Biological replication**	**The seeding percentage of Mak-f, %**	**Actual percentage of Mak-f, %**	**Change in chimeric iLOs area after 24 h of incubation with forskolin, mean value ± SD**	**p-value**
GFP-positive Mak-f (CFTR WT/WT) + P1L5 (CFTR F508del/F508del)	1st	0	0	1.03 ± 0.06	-
		1	1	1.09 ± 0.05	0.1697
		5	10	1.2 ± 0.08	0.0005
		10	39	1.32 ± 0.09	<0.0001
		15	55	1.4 ± 0.1	<0.0001
		20	65	1.36 ± 0.1	<0.0001
		100	100	1.43 ± 0.04	<0.0001
	2nd	0	0	1.05 ± 0.06	-
		1	17	1.15 ± 0.07	0,0084
		5	23	1.16 ± 0.06	0,0014
		10	52	1.23 ± 0.09	0.0001
		15	62	1.35 ± 0.16	0.0002
		20	71	1.38 ± 0.09	<0.0001
		100	100	1.58 ± 0.06	<0.0001
	3rd	0	0	1.13 ± 0.04	-
		1	1	1.15 ± 0.05	0.6276
		5	7	1.15 ± 0.06	0.7072
		10	27	1.2 ± 0.07	0.0247
		15	61	1.29 ± 0.08	<0.0001
		20	73	1.25 ± 0.05	<0.0001
		100	100	1.4 ± 0.05	<0.0001
FP-positi Mak-f (CFTR /T/WT) [illegible]	1st	0	0	1.08 ± 0.09	-
		1	18	1.24 ± 0.15	0.0423

		5	36	1.23 ± 0.06	0.0005
		10	62	1.52 ± 0.28	0.0006
		15	75	1.57 ± 0.17	0.0006
		20	92	1.61 ± 0.27	0.0001
		100	100	1.67 ± 0.15	<0.0001
	2nd	0	0	1.11 ± 0.08	-
		1	4	1.13 ± 0.14	0.9992
		5	8	1.16 ± 0.16	0.939
		10	65	1.29 ± 0.09	0.0032
		15	72	1.37 ± 0.1	0.0001
		20	82	1.38 ± 0.14	0.0006
		100	100	1.38 ± 0.09	<0.0001
	3rd	0	0	1.11 ± 0.08	-
		1	3	1.17 ± 0.15	0.8849
		5	10	1.3 ± 0.19	0.0406
		10	22	1.34 ± 0.19	0.0179
		15	28	1.31 ± 0.15	0.0019
		20	75	1.36 ± 0.05	<0.0001
		100	100	1.34 ± 0.07	<0.0001

Swelling kinetics and the choice of the 24 h endpoint

Material and methods

To identify the imaging time at which the forskolin-induced swelling assay best discriminates between CFTR-competent fractions, the interval from 0 to 24 h after forskolin addition was examined with images acquired every 2 h. Chimeric iLOs were assembled at three conditions — 0% Mak-f (P1L5 only), a planned 15% Mak-f fraction and 100% Mak-f — and passaged onto a single 96-well plate, twelve wells per condition, as described in Section 4.8 of the main text. Forskolin was added to a final concentration of 10 µM, and bright-field images were acquired on a Lionheart FX automated imager at 4× magnification immediately before stimulation (t = 0 h) and every 2 h thereafter up to 24 h, giving thirteen timepoints. Images were processed with the same semi-automated segmentation, artifact-removal and tracking algorithm used for the endpoint experiments (Section 4.9 of the main text), and the area ratio at each timepoint was calculated as the 2D projected area of each tracked organoid divided by its own area at t = 0 h.

Because this experiment comprised a single unreplicated plate, it was analysed descriptively and was not included in the linear mixed-effects model or in the ROC analysis.

Results

Swelling increased monotonically in all three conditions throughout the 24 h window and had not reached a plateau at the final timepoint (Figure S6). At 8 h the geometric mean area ratio was 1.198 ± 0.189 in the 100% Mak-f condition, 1.156 ± 0.172 in the chimeric condition and 1.043 ± 0.063 in the 0% Mak-f condition; at 24 h the corresponding values were 1.463 ± 0.286, 1.322 ± 0.206 and 1.195 ± 0.239. The absolute difference between the 100% and the 0% condition increased from 0.156 at 8 h to 0.268 at 24 h, and between the chimeric and the 0% condition from 0.113 to 0.127. The dispersion between organoids within a condition increased over the same interval, and the differences expressed as multiples of the pooled within-condition standard deviation were not larger at the end of the window than in the middle of it (100% against 0%: 1.1 at 8 h and 1.0 at 24 h; chimeric against 0%: 0.9 at 8 h and 0.6 at 24 h). The data therefore show that the response continues to grow in absolute terms up to 24 h, but do not show that 24 h separates the conditions better than an earlier timepoint. 24 h was selected as the endpoint for all FIS measurements in this study as the longest interval examined, at which swelling was still rising in every condition.

The absolute area ratios of this experiment should not be compared directly with those of the endpoint experiments. The 0% Mak-f condition reached 1.195 ± 0.239 at 24 h, whereas the CF-only condition means of the six endpoint experiments lie between 1.03 and 1.13 (Table S5). The two sets of values are not the same quantity: those reported here are geometric means taken directly across all tracked organoids of a condition, and only organoids segmented and tracked at all thirteen timepoints contribute, whereas the endpoint values are condition means of well-level geometric means and require a pair of images only. The present experiment also differed in handling, the plate being removed from the incubator and imaged thirteen times over 24 h, and it was run once, on a single plate; a genuinely higher baseline on this plate cannot be separated from these differences. The experiment was accordingly analysed descriptively and was not included in the linear mixed-effects model or in the ROC analysis, and the area-ratio cutoffs of 1.16 and 1.21, derived from well-level data of the endpoint experiments, do not apply to the organoid-level values reported here.

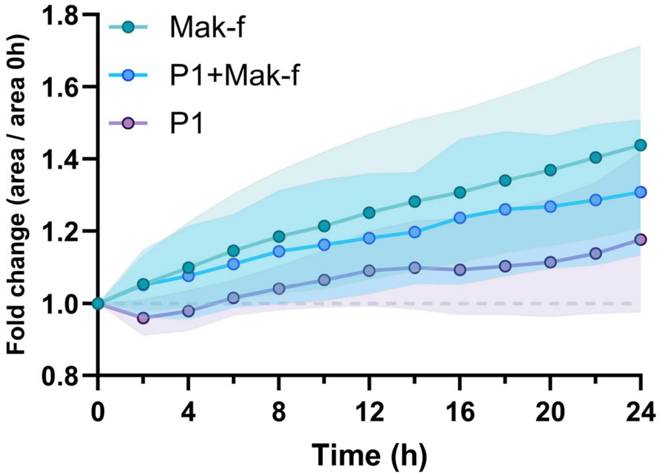

Figure S6. Swelling kinetics of chimeric iLOs over 24 h of forskolin stimulation. Chimeric iLOs were assembled at three conditions — 100% Mak-f (*CFTR* WT/WT-GFP), a planned 15% Mak-f fraction (85% P1L5 *CFTR* F508del/F508del + 15% Mak-f) and 0% Mak-f (P1L5 only) — passaged onto a single 96-well plate, stimulated with 10 µM forskolin and imaged in bright field every 2 h from t = 0 h to t = 24 h, giving thirteen timepoints. For each organoid the area ratio at a given timepoint is its 2D projected area at that timepoint divided by its own area at t = 0 h. Symbols, geometric mean of the per-organoid area ratios; shaded band, ± 1 SD. n = 170 organoids per condition, tracked across all thirteen timepoints, from 12 wells per condition on one plate; the experiment was not replicated across plates. Unlike the endpoint experiments, where the well is the unit of analysis, values here are geometric means of the per-organoid area ratios taken directly across all tracked organoids of a condition and are not directly comparable with the area ratios reported in the main text or in Table S5.
